# Genome-Wide Comprehensive Survey of the Subtilisin-Like Proteases Gene Family Associated With Rice Caryopsis Development

**DOI:** 10.3389/fpls.2022.943184

**Published:** 2022-06-20

**Authors:** Kaifeng Zheng, Lu Pang, Xiuhua Xue, Ping Gao, Heping Zhao, Yingdian Wang, Shengcheng Han

**Affiliations:** ^1^Beijing Key Laboratory of Gene Resources and Molecular Development, College of Life Sciences, Beijing Normal University, Beijing, China; ^2^Academy of Plateau Science and Sustainability of the People’s Government of Qinghai Province and Beijing Normal University, Qinghai Normal University, Xining, China

**Keywords:** subtilisin-like proteases, gene family, expression in rice caryopsis, *Oryza*, rice

## Abstract

Subtilisin-like proteases (SUBs), which are extensively distributed in three life domains, affect all aspects of the plant life cycle, from embryogenesis and organogenesis to senescence. To explore the role of SUBs in rice caryopsis development, we recharacterized the *OsSUB* gene family in rice (*Oryza sativa* ssp. *japonica*). In addition, investigation of the *SUBs* was conducted across cultivated and wild rice in seven other *Oryza* diploid species (*O. brachyantha*, *O. glaberrima*, *O. meridionalis*, *O. nivara*, *O. punctata*, *O. rufipogon*, and *O. sativa* ssp. *indica*). Sixty-two *OsSUBs* were identified in the latest *O. sativa* ssp. *japonica* genome, which was higher than that observed in wild species. The *SUB* gene family was classified into six evolutionary branches, and SUB1 and SUB3 possessed all tandem duplication (TD) genes. All paralogous *SUBs* in eight *Oryza* plants underwent significant purifying selection. The expansion of *SUBs* in cultivated rice was primarily associated with the occurrence of tandem duplication events and purifying selection and may be the result of rice domestication. Combining the expression patterns of *OsSUBs* in different rice tissues and qRT–PCR verification, four *OsSUBs* were expressed in rice caryopses. Moreover, *OsSUBs* expressed in rice caryopses possessed an earlier origin in *Oryza*, and the gene cluster formed by *OsSUBs* together with the surrounding gene blocks may be responsible for the specific expression of *OsSUBs* in caryopses. All the above insights were inseparable from the continuous evolution and domestication of *Oryza*. Together, our findings not only contribute to the understanding of the evolution of SUBs in cultivated and wild rice but also lay the molecular foundation of caryopsis development and engineering improvement of crop yield.

## Introduction

Cultivated rice stands out as one of the most important food crops in the world, and in the genus *Oryza*, two species were independently domesticated and culminated as *Oryza glaberrima* and *Oryza sativa* (Chen et al., 2019). Currently, the interests of many rice breeders converge on cultivars with higher yields and better health. By modulating four major factors affecting rice yield (grain weight, grain numbers per panicle, panicle numbers per plant, and ratio of filled grains), newly bred “super” rice cultivars have the opportunity to emerge (Sakamoto and Matsuoka, 2008; Yang and Zhang, 2010). The caryopses of different positions on the panicle have been demonstrated to primarily result in discrepancies in weight grain and filling rate. Generally, caryopses on the proximal secondary branch (CSB) hold smaller grain sizes due to their lower proliferation rates and poorer filling rates of endosperm compared to caryopses located on the primary apical branch (CPB; Yang et al., 2000; Ishimaru et al., 2003). More importantly, newly bred “super” rice cultivars exhibit a noticeable developmental difference between CPB and CSB (Yang and Zhang, 2010). Consequently, further investigating the developmental molecular enigma in caryopses, especially in CPB and CSB, can lay a solid foundation for breeding increased high-yielding and high-quality cultivated rice.

A double fertilization event in the plant ovule symbolizes the beginning of caryopsis development, while a dormant seed indicates the terminus of fruit development. During the developmental stage after fertilization, in addition to forming the embryo, the caryopsis accumulates the endosperm, which ultimately determines grain size and weight. The development progress of the endosperm is determined by two successive stages: early-stage endosperm cell proliferation and differentiation and late-stage grain filling (Olsen, 2004). Synthase (SUS), UDP-glucose pyrophosphorylase (UGPase), and other enzymes have been characterized to finely normalize starch biosynthesis and accumulation. The low activity of these enzymes has also been associated with poor grain filling in CSB compared to CPB (Tang et al., 2009). In addition, various endogenous phytohormones and external environmental conditions drive homeostasis in the filling of grain (Xu et al., 2007; Yang et al., 2006; Chang et al., 2020). The seed size and weight of individual spikelets are affected by protein modifications, such as ubiquitination and histone acetylation; G protein-coupled, MAPK, and other classical signal transduction processes are also involved in the regulation of sink capacity (Weng et al., 2008; Song et al., 2015; Sun et al., 2018; Xu et al., 2018). Using the transcriptome and metabolome, the differential expression of starch synthesis and hormone-related genes between CPB and CSB was shown to occur during the later stage of grain filling, and differences in some miRNAs also indicated differential developmental mechanisms between CPB and CSB (Zhu et al., 2011; Wang et al., 2022).

The catalytic class of serine peptidases has become a unique protease in plants (Barrett and Rawlings, 1995; García-Lorenzo et al., 2006). Subtilisin-like proteases (SUBs or SBTs), making up the S8 peptidase family, form the largest group of serine peptidases and are present in all three major life domains (Siezen et al., 1991; Rawlings et al., 2008). Most subtilisins in plants are synthesized as inactive preprotein precursors and consist of several conserved functional domains, such as the peptidase S8 domain, protease-associated (PA) domain, and inhibitor I9 domain. These functional and structural domains are closely associated with acceptable structural transformation (displacement of the Ser from the catalytic triad, homodimerization of the protein) and functional exertion (protein–protein interactions, intramolecular chaperone; Siezen and Leunissen, 1997; Figueiredo et al., 2018; Schaller et al., 2018). Plant subtilases with apparent Ca^2+^-independence and glycosylation are also involved in plant responses, programmed cell death, and plant–pathogen interactions (Bykova et al., 2006; Ottmann et al., 2009; Fernández et al., 2014). On the cell wall, *SUBs* modulate the structural characteristics of the cell wall and extracellular signaling molecule activity to regulate development. Regarding the phylogeny of *SUBs* and family organization, numerous studies on the *SUB* gene family in plants have been published, including 55 members from *Arabidopsis thaliana*, 82 members from *Vitis vinifera*, 82 members from tomato, and 63 members from *O. sativa* ssp. *japonica* (Rautengarten et al., 2005; Tripathi and Sowdhamini, 2006; Figueiredo et al., 2016; Norero et al., 2016). In total, *SUBs* impact all phases of the plant life cycle, from embryogenesis and organogenesis to senescence and programmed cell death.

In recent works, we analyzed the transcriptome-wide gene expression of CPB and CSB in four different stages after flowering to obtain *Os07g39020* (*OsSUB5*3), encoding subtilisin-like proteases, which are differentially expressed between CPB and CSB (Chang et al., 2020). A prior study reported preliminary identification of the subtilisin family in *O. sativa* ssp. *japonica* (Tripathi and Sowdhamini, 2006). However, the rice genome has been continuously finely sequenced, and annotation information has been updated (Jackson, 2016; Wing et al., 2018; Kong et al., 2019). Therefore, the reidentification and analysis of the *SUB* gene family in rice is essential to further elucidate the unique developmental discrepancy between CPB and CSB. Furthermore, across cultivars and wild species, we also broadened the survey of the *SUBs* to seven other *Oryza* species (*O. brachyantha*, *O. punctata*, *O. meridionalis*, *O. glaberrima*, *O. nivara*, *O. rufipogon*, *O. sativa* ssp. *indica*). In conclusion, we not only elucidated the evolutionary history of *SUBs* across cultivars and wild *Oryza* but also clarified that *SUBs* may be a critical factor in determining the developmental mechanisms in rice caryopses.

## Materials and Methods

### Plant Materials and Growth Conditions

Rice plants (*O. sativa* spp. *japonica* cv. Zhonghua11, ZH11) were grown under natural conditions from May to October annually in a field at Beijing Normal University (Beijing, China). In July, CPB and CSB were collected at 0, 3, 5, and 12 days after flowering (DAF). They were rapidly conducted in liquid nitrogen, and frozen at −80°C to store for a long time.

### Identification and Retrieval of *SUB* Family Members in the *Oryza* Genus

We downloaded the whole genome and annotation data of eight species in *Oryza*, which are publicly available in Ensembl Plants (http://plants.ensembl.org/index.html, accessed on September 2021) and the Rice Genome Annotation Project (RGAP; http://rice.uga.edu/index.shtml, accessed on September 2021). The eight species of *Oryza* are shown in Supplementary Table 1, with their Assembly Name and Accession Number. TBtools^1^ was utilized as the data analysis and visualization software. We used the protein sequences of 55 AtSUBs (except AtSBT4.10) to BLAST against eight species (E-value = 1e-5; number of His = 500; number of alignments = 250; Tripathi and Sowdhamini, 2006). Then, we deleted sequence redundancies and employed BLAST^2^ on the UniProtKB/Swiss-Prot (SwissProt) database. The peptidase S8 domain, protease-associated (PA) domain, and inhibitor I9 domain were checked using the CD-Search Tool.^3^

### Multiple Sequence Alignment and Phylogenetic Analysis

A total of 477 SUB protein sequences were pooled from eight different *Oryza* plants and *A. thaliana* (Supplementary Table 2) and submitted to MAFFT^4^ (version 7) for multiple alignments (Ouyang et al., 2007; Rozewicki et al., 2019). Furthermore, to observe the evolutionary relationships of SUB proteins in *O. sativa* ssp. *japonica*, we individually selected 62 OsSUBs for sequence alignment and phylogenetic analysis. The neighbor-joining tree (N-J tree) was constructed using MEGAX^5^ (Bootstrap Number = 1,000; gaps/missing data treatment = partial deletion; Site Coverage Cut-off = 85; Kumar et al., 2018). To improve the readability and aesthetics of the evolutionary tree, the resulting N-J Tree was edited using FigTree^6^ (version 1.4.4).

### Chromosomal Distribution and Duplication Analysis

Combining rice gene location information, all family members of *OsSUBs* were mapped to their corresponding chromosomes. Moreover, we focused on *SUB* tandem duplication and collinearity events. We conducted self-BLAST using whole-protein files from rice (E-value = 1e-3; number of His = 10), and the gene location data were analyzed using MCScanX.^7^ To compare the tandem duplication (TD) status of *SUBs* in the *Oryza* species, we manually analyzed each genome.

### Ka/Ks Calculation and Selection Survey

First, we investigated the selection pressure of tandem duplication (TD) genes. The “Simple Ka/Ks Calculator” module was utilized to calculate the Ka (nonsynonymous substitution rate) and Ks (synonymous substitution rate) of TD *SUBs*. The Ka/Ks ratio characterizes the selection pressure of *SUBs*. Subsequently, using the Coden-Based Z-test in MEGAX, the Test Hypothesis was set to Purifying Selection, Positive Selection, and Neutral Selection (Partial Deletion; Site Coverage Cut-off = 80). We examined all the *SUB* family members in each species for overall average and in sequence pairs. Here, *p* < 0.05 indicated significant selection.

### Conserved Protein Domain and Gene Structure Analysis

To identify the presence of Peptidases_S, the Inhibitor I9 domain, the Fibronectin (Fn)-III domain, and other typical functional domains in OsSUB proteins, we used the CD-Search Tool and chose Pfam-18,271 PSSMs (Lu et al., 2020). Considering the importance of gene structure features in gene families, the structural characteristics of 62 *OsSUBs* in rice were examined. We used the currently published gene structure annotation to describe intron/exon structure.

### Expression Pattern Analysis of *OsSUBs* in Different Developmental Stages and Tissues

To further explore the existing patterns of *OsSUBs* in different rice tissues and development stages, we obtained an RNA-seq library using RGAP. The Rice Expression Matrix File in the RNA-seq libraries from Nipponbare was derived from the NCBI Sequence Read Archive (SRA). Subsequently, we visualized the data using heatmaps (Log Scale: Base = 2; LogWith = 1). We conducted column standardization to investigate the expression status of the same gene in different developmental stages.

### Total RNA Extraction and Quantitative Real-Time PCR

Here, we focused on *OsSUB29*, *OsSUB53*, *OsSUB58* and *OsSUB63*, while *OsASP1* was used as a control (Chang et al., 2020). Total RNA from caryopses at different developmental stages was extracted using TRIzol^®^ reagent according to the manufacturer’s protocol (Invitrogen, United States). Combined with the PureLink^®^ DNase kit (Invitrogen), we used the PureLink^®^ RNA Mini Kit (Invitrogen) to purify total RNA. To generate cDNA, approximately 2 μg of total RNA was reverse-transcribed utilizing the Reverse-Aid^™^ First Strand cDNA Synthesis Kit (Fermentas, Canada). Using PCR Master Mix (Power SYBR^®^ Green; Applied Biosystems, United States), we conducted qRT–PCR on a 7,500 Fast qRT–PCR System (Applied Biosystems). The thermal cycle was 2 min at 50°C, 10 min at 95°C, followed by 40 cycles of 15 s at 95°C and 60 s at 60°C. We examined the target amplification product specificity using a dissociation curve program. We performed three independent biological replicates of qRT–PCR, and *OsActin3* was used as an internal control. |A fold change in the relative gene expression value| ≥ 2 indicates a significant difference between any two samples. The primers are shown in Supplementary Table 3.

### Synteny Analysis of *Oryza* Genomes and *OsSUBs*

To obtain a preliminary synteny relationship of *SUBs* between wild and cultivated rice, we utilized the whole genome from Ensembl Plants. Wild and cultivated rice species were examined using MCScanX software (minimum chain length of collinear genes = 30; Num BlastHits = 10; E-value = 1e-3). To explore the evolutionary history of several *OsSUBs* (*OsSUBs29*, *OsSUBs53*, *OsSUBs58*, and *OsSUB63*), synteny of the genomic neighborhood, including orthologues or paralogues, was obtained from Genomics (database version 105.01)^8^.

## Results

### Domesticated Rice Possesses More *SUB* Genes in Reidentification

**Figure 1:**
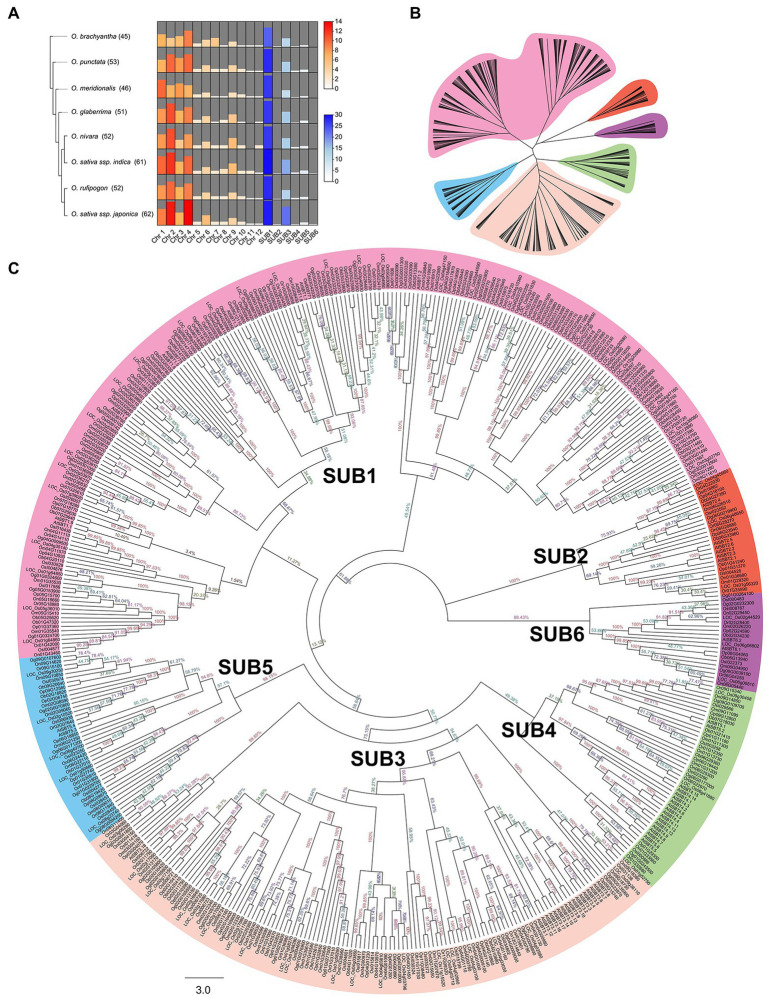
Identification and phylogenetic relationships of SUBs in eight *Oryza* species. **(A)** Comparison of the number of *SUB* gene family members in eight *Oryza*. The red section of the heatmap reflects the number variation of *SUBs* on twelve chromosomes. The blue portion of the heatmap visualizes the number of *SUBs* within the six clades. The height of the bars and the shade of the color indicate the size of the numbers. **(B)** An abbreviated evolutionary tree clearly shows the six primary separated groups and is labeled with different color backgrounds. **(C)** After performing alignment of 477 subtilases protein sequences, a phylogenetic tree of SUBs was produced using the neighbor-joining method. According to the classification, the branches of different clades are indicated by various colors, and the bootstrap values are noted at each branch node.

Blasted with 55 AtSUB protein sequences, we collected 422 putative subtilisin genes from three domesticated species (*O. glaberrima, O. sativa* ssp. *indica*, and *O. sativa* ssp. *japonica*) and five wild species (*O. brachyantha*, *O. meridionalis*, *O. nivara*, *O. punctata*, *O. rufipogon*). The accession number of each *SUB* and the basic properties of the proteins are summarized in Supplementary Table 2. The number of specific family members in each species and the number of *SUBs* on each chromosome were visualized in a heatmap, and chromosomes 2 and 4 contained most of the family members (Figure 1A).

Lineage-specific expansion or contraction of gene families indicated the evolution of genes. Interestingly, the more domesticated the *Oryza* plants, the more *SUB* family members they possessed, especially on chromosomes 2 and 4 (Supplementary Table 2; Figure 1). More than 60 *SUBs* were identified in the domesticated and cultivated rice (*O. sativa*), which was more than previously identified in any other species, especially the wild rice *O. brachyantha* (45 members). Due to habitat and climate, *O. meridionalis* also exhibited a status of wild rice in terms of its small number of *SUBs* (46 members). These results imply that domesticated rice contain more members of the *SUB* gene family.

Tripathi and Sowdhamini (2006) performed a preliminary identification indicating that *O. sativa* ssp. *japonica* contained 63 *SUB* members. Although we identified 62 members in rice, this was slightly different from previous results. We found that LOC_Os01g17160 (*OsSUB1*) did not encode proteins with the functional domains of subtilisin, such as Peptidases_S, and was excluded as a member of the *SUB* family. Notably, after manual inspection, almost all SUB proteins in *Oryza* contained the above characteristic architectural domains. Comparing these results to previous information, five members of the *OsSUBs* were updated, one was removed, and one was appended (Supplementary Table 4).

### The *SUB* Gene Family in *Oryza* Is Divided Into Six Major Clusters

To compare the evolutionary relationships of SUBs in several *Oryza* species, phylogenetic analyses of 422 SUB proteins were performed using the neighbor-joining method. The consensus phylogeny is shown in Figure 1, which contains an abbreviated evolutionary tree that better shows the different clades (Figure 1B). In the dendritic evolutionary tree shown in Figure 1, the node and position relationships of the six clusters are visible. We divided the SUBs into six groups, classifying them as SUB1 to SUB6 (Figures 1B,C). SUB1 and SUB3 were the two most prominent subclasses, and the gene family number expansion accompanying species domestication was primarily observed in SUB3. On the other hand, the groups of SUBs in *Oryza* were the same as the six major clades of *A. thaliana*. Two subspecies of cultivated rice, *O. sativa* ssp. *japonica* and *O. sativa* ssp. *indica*, had a closer genetic relationship than the wild rice *O. rufipogon*, while *O. brachyantha* had a more distant evolutionary relationship.

### The *OsSUB* Gene Family, Distributed on 12 Chromosomes, Has Collinearity and Duplications in Several Genes

**Figure 2:**
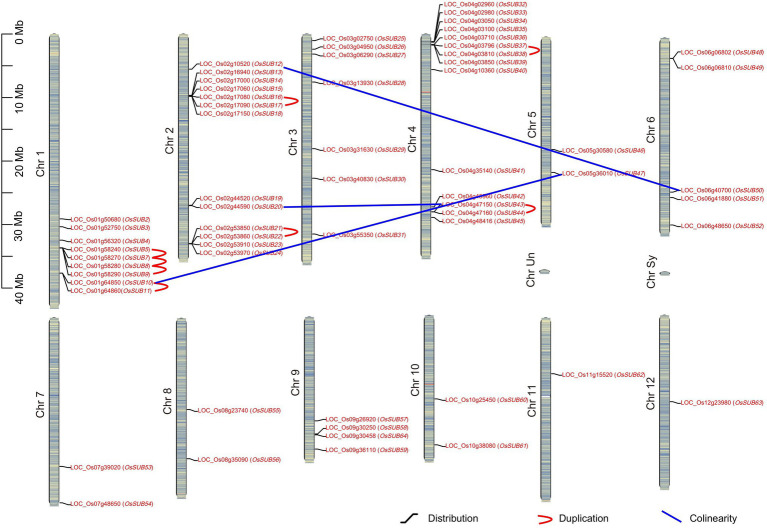
Chromosome distribution, duplication, and colinearity of *SUB* genes in rice. Diagram of twelve chromosomes of *O. sativa* ssp. *japonica* is depicted, and 62 *OsSUBs* are mapped to the chromosomes. Tandem duplications are shown using a red curve, and blue lines connect genes, which hold a collinearity relationship. The heatmap on the chromosome roughly depicts the rice chromosome gene density.

The chromosome distribution of *SUBs* in *O. sativa* ssp. *japonica* is shown in Figure 2, and the *OsSUBs* covered 12 chromosomes. Generally, more *SUBs* mapped to chromosomes 1, 2, 3, and 4, while chromosomes 5, 7, 8, 10, 11, and 12 carried only one or two *SUB* genes. If a species has undergone chromosomal polyploidization or rearrangements during its evolution, regions of collinearity could be found in its genome. Furthermore, we observed collinearity in three pairs of genes (*OsSUB10* and *OsSUB47*; *OsSUB20* and *OsSUB43*; and *OsSUB12* and *OsSUB50*). *OsSUB20* and *OsSUB12* are positioned on chromosome 2, while *OsSUB43* and *OsSUB50* are localized on chromosome 4. *OsSUB10* and *OsSUB47* appeared on chromosomes 1 and 5, respectively. Additionally, *O. sativa* ssp. *japonica* exhibited tandem duplication (TD) events of *SUBs*. Four genes, *OsSUB5*, *OsSUB7*, *OsSUB8*, and *OsSUB9,* on chromosome 1 constituted an obvious tandem replication block (Figure 2). The N-J tree showed that these four genes were paralogous, so segment duplication played a specific role in expanding the SUB3 class of *the OsSUB* gene family (Figure 1). Five pairs of genes (*OsSUB10* and *OsSUB11*; *OsSUB16* and *OsSUB17*; *OsSUB21* and *OsSUB22*; *OsSUB37* and *OsSUB38*; and *OsSUB43* and *OsSUB44*) also possessed tandem duplication relationships in the *OsSUB* gene family.

### Tandem Duplication Genes of the *SUB* Family Exist in the SUB1 and SUB3 Clusters Under Purifying Selection

To better understand the role of tandem duplications (TDs) and the expansion of *SUBs*, we investigated the remaining seven *Oryza* species in our analysis (Table 1). We confirmed that *SUB* tandem repeats almost exclusively occurred on chromosomes 1, 2, and 4. However, there was one *SUB* duplication event on chromosome 7 in the ancient ancestral wild species *O. brachyantha*, which possessed the fewest number of tandem duplicated gene pairs. In contrast, the high frequency of tandem duplicated *SUBs* in two cultivated rice species, *O. sativa* ssp. *japonica* and *O. glaberrima*, suggesting that TD events may motivate the expansion of the *SUB* gene family. It cannot be ignored that the TD of *SUBs* only occurred in clades 1 and 3. This means that the formation of SUB1 and SUB3, two clades containing the most family members, is closely related to the TD event.

**Table 1:** Tandem duplication (TD) of *SUBs* in eight *Oryza* species.

Species	Chr	Collinearity gene pair	Clade	Ka/Ks (Average)
*O. sativa ssp. japonica*	1	*OsSUB5*, *OsSUB7*, *OsSUB8*, *OsSUB9*	3	0.1714
	1	*OsSUB10, OsSUB11*	1	0.7775
	2	*OsSUB16, OsSUB17*	3	0.3293
	2	*OsSUB21, OsSUB22*	1	0.4642
	4	*OsSUB37, OsSUB38*	3	0.2806
	4	*OsSUB43, OsSUB44*	1	0.7184
*O. sativa* ssp. *indica*	1	OsI004600, OsI004601	3	0.2267
	2	OsI006724, OsI006725, OsI006722, OsI006721	3	0.2316
*O. rufipogon*	1	Or01G37000, Or01G37010	3	0.1688
	2	OrO2G11750, OrO2G11760	3	0.4579
	2	Or02G36200, Or02G36210	1	0.7041
	4	Or04G22980, Or04G22990	1	0.2102
*O. nivara*	2	On02G13150, On02G13160, On02G13170	3	0.2131
	2	On02G36760, On02G36770	1	0.4342
	4	On04G00780, On04G00790	3	0.3043
	4	On04G19920, On04G19930	1	0.7204
*O. glaberrima*	1	Og01G0276900, Og01G0277000	3	0.2172
	2	Og02G0105600, Og02G0105700, Og02G0105800, Og02G0105900	3	0.2748
	2	Og02G0300100, Og02G0300200	1	0.4667
	4	Og04G0009000, Og04G0009100	3	0.2295
*O. meridionalis*	1	Om01G35530, Om01G35540	1	0.7463
	2	OmO2G12330, OmO2G12340	3	0.2770
	4	OmO4G17870, OmO4G17880	1	0.7597
*O. punctata*	2	Op02G10390, Op02G10400, Op02G10410, Op02G10420	3	0.1939
	2	Op02G32020, Op02G32030, Op02G32040	1	0.4530
	4	Op04G19090,Op04G19100	1	0.6365
*O. brachyantha*	1	Ob01G42570, Ob01G42580	3	0.1344
	7	Ob07G25630, Ob07G25640	1	0.4326

When the ratio of Ka/Ks was >1, <1, or = 1, we thought that positive selection, negative selection (purify selection), or neutral selection had occurred among genes, respectively. Since tandem duplication of genes can suggest that genes are subject to selection and environmental stress, we examined the occurrence of positive selection of TD gene pairs in eight *Oryza*. All results for Ka/Ks were less than 1, meaning that all paralogous homologous genes were under purifying selection, not positive selection (Table 1). We then calculated their mean values for the number of tandem repeat genes exceeding two. Notably, the mean Ka/Ks of all TD genes in clade 3 was 0.2434, while the value in clade 1 was 0.5246, and all Ka/Ks values higher than 0.7 were found in clade 1. Compared to SUB1, SUB3 was subject to more robust purifying selection.

### *SUBs* From *Oryza* Generally Underwent Significant Purifying Selection Rather Than Positive Selection

**Figure 3:**
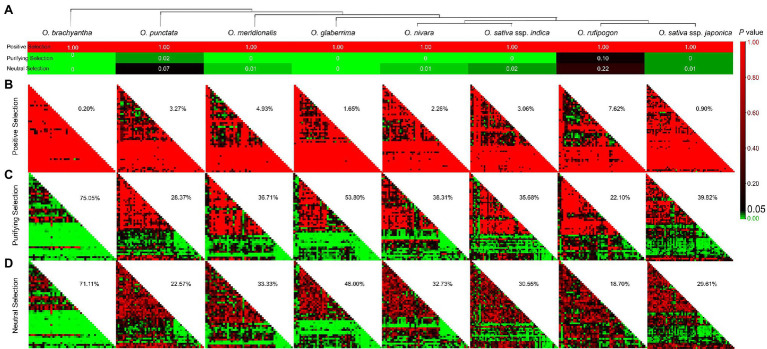
Adaptive evolutionary profiles correspond to the *SUB* gene family in *Oryza.* **(A)** Overall average of the *SUB* family in eight species by the Coden-Based Z-test. **(B–D)** Value of *p* matrix for positive selection, purifying selection, and neutral selection. We considered *p* < 0.05 to indicate significant selection. In each *Oryza* plant, percentages indicate the extent to which significantly selected loci account for the total.

After clarifying that all TD *SUBs* were subjected to purification selection, Ka/Ks of *SUBs* in each *Oryza* were calculated, and a Coden-Based Z-test was performed. The value of *p* is shown after the overall average analysis in Figure 3A. All *p* = 1 in the positive selection assay, so we cannot absolutely reject the hypothesis that it was strictly neutral evolution and not positive selection. Following a purification screen, we obtained *p* < 0.05, except for *O. rufipogon*, for which we tentatively accepted negative selection and rejected neutral evolution. In contrast, when the neutral selection hypothesis (Ka = Ks) was examined (*p* < 0.05), except for *O. punctata* and *O. rufipogon*, we rejected neutral evolution. When investigating positive selection on the overall average, the results suggested that *SUBs* in *Oryza* may have experienced purifying selection.

Subsequently, we analyzed adaptive selection among individual gene pairs and separately calculated the number of *p* < 0.05 as a percentage of the total number within the matrix, which allowed a rough comparison of the selection status of gene families in different species. Value of *p* < 0.05 indicated that two sequences underwent significant positive selection after divergence, and *O. rufipogon* had the highest positive selection ratio of 7.62% (Figure 3B). Figures 3C,D shows that some *SUBs* of the eight species were subjected to both purifying and neutral selection, with *p* < 0.05 implying significance. However, purifying selection contrasted with a higher proportion of significance (Figures 3C,D). At this point, we tentatively concluded that the *Oryza SUBs* were predominantly purifying selection, and the *SUB* gene family was still relatively stable. Combined with the evolutionary tree of the species, we found that the ancestral species *O. brachyantha* experienced a broader range of purifying selection. In contrast, cultivated rice (*O. sativa*), especially the *O. sativa* ssp. *japonica* (27.85%), experienced a smaller range of negative selection. We speculated a smaller purification ratio in the more domesticated cultivated rice, but there were still some values that did not match our speculation. The purification ratio was more minor in wild rice *O. punctata* (28.37%) and 53.80% in the West African cultivated rice *O. glaberrima*.

### Some *OsSUBs* Share Similar Protein Domains but With Different Gene Structures

**Figure 4:**
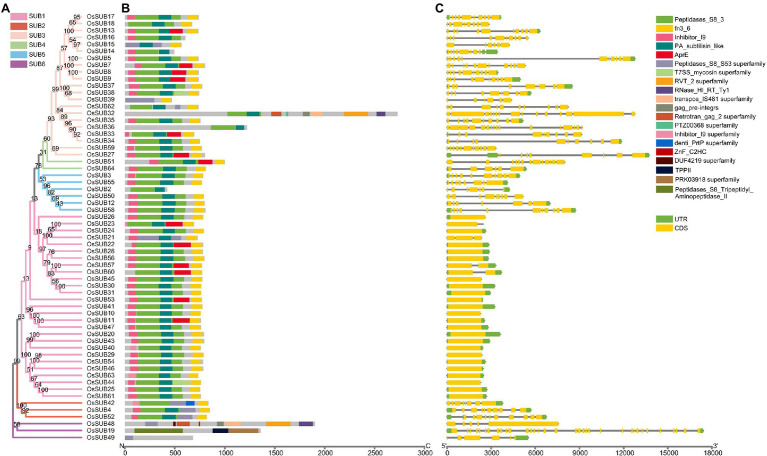
Detailed information on the phylogenetic relationships, protein domains and gene structures of *OsSUBs*. **(A)** Phylogenetic tree of the 62 SUB proteins in *O. sativa* ssp. *japonica*. The N-J tree of OsSUBs is colored using the color scheme in Figure 1C, and the bootstrap values are indicated at the branch nodes. **(B)** Conserved functional structural domains of OsSUB proteins. **(C)** The horizontal black line, yellow box and green box indicate the position of introns, CDS, and UTR, respectively. The scale bar below shows the nucleotide length of 18,000 bp.

To gain insight into the *OsSUBs* and their encoded proteins, we built a phylogenetic tree of the 62 OsSUB proteins and presented the functional domains together with their corresponding gene architectures (Figure 4). According to the N-J tree, the 62 protein sequences were classified into six clades, consistent with the phylogenetic classification relationships presented in Figure 1. Excluding SUB6, which has high homology with mammals, almost all proteins in the remaining five clades contained four primary conserved domains (Peptidases_S, Inhibitor I9, Fibronectin (Fn)-III, and Protein Associated domain (PA) subtilisin-like). Nevertheless, the AprE structural domain, a posttranslational modification, and chaperones appeared in all clades except SUB5 and 6 (Figure 4B).

The SUB1 clade had many family members and comprised twenty-seven *OsSUB* genes with fewer introns from chromosomes 1, 2, 3, 4, 5, 7, 8, 9, 10, and 12 (Figure 4C). The gene length of SUB1 group members was smaller than that of the members from the rest of the SUB clades, but the length did not vary greatly. The SUB2 clade included three *OsSUBs* mapping to three chromosomes. Upstream of the Fn-III domain, the Peptidases_S8_S53 superfamily was present and served as a marker to distinguish the SUB2 clade. The role of exon/intron diversification events of gene family members in the evolution of multigene families cannot be ignored (Xu et al., 2012). However, the SUB3 clade was characterized by a great diversity of gene structures and lengths, such as a massive number of introns and extensive segments of introns. The CDS architecture of the four tandem duplication genes, *OsSUB5*, *OsSUB7*, *OsSUB8*, and *OsSUB9*, was very similar, and the expansion of introns was quite evident. Figure 3C shows that gene structure changes in the SUB3 clade were associated with intron retention and CDS expansion (*OsSUB32*). Based on our phylogenetic analysis, the SUB4 clade was the smallest cluster with only two gene family members, which was distinct from the more than a dozen *AtSUBs*. Six *OsSUBs* occurred in the SUB5 clade, with *OsSUB12* and *OsSUB50* having collinearity relationships. Four *OsSUBs* appeared in this group with direct homology to the mammalian V_clade. The most striking example was the tripeptidyl peptidase II (TPP2) structural domain in both *OsSUB19* and *AtSBT6.2* protein sequences. *OsSUB19* was the gene with the highest number of introns and the longest total gene length among the 62 genes. Taken together, we found gene members that consistently encoded similar proteins, which tended to be similar in length, structural domain type, and distribution relationships. However, the gene structures of these members were very diverse, and they were often associated with the type of cluster.

### Only *OsSUB29*, *OsSUB53*, *OsSUB58*, and *OsSUB63* Are Specifically Expressed in 5 DAP Seeds

**Figure 5:**
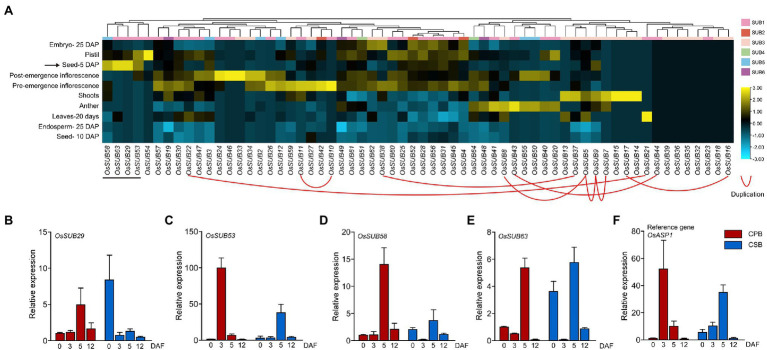
Tissue-specific expression of the *OsSUB* gene. **(A)** Expression patterns of the *OsSUB* gene family in different rice tissues and developmental stages. The scale indicating the relative signal strength values is displayed on the right side of the heatmap (Log Scale: Base = 2; LogWith = 1). Genes with tandem duplication relationships are linked to each other with red lines. **(B–F)** The expression profiles of four *OsSUBs* and *OsASP1* were determined using quantitative real-time polymerase chain reaction (qRT–PCR) in CPBs at 0, 3, 5, and 12 days after heading (DAH; CPB-0, −3, −5, and −12) and CSBs at 0, 3, 5, and 12 DAH (CSB-0, −3, −5, and −12). We performed three independent biological replicates, and *OsActin3* was used as an internal control.

First, we combined the tissue expression profiles with TD gene pairs within the *OsSUB* gene family and found that only *OsSUB5*, *OsSUB7*, and *OsSUB9* shared similar expression patterns (Figure 5A; Supplementary Table 5). The other paralogous genes exhibited different expression profiles among themselves, implying that the duplicated genes may not share the same molecular functions. Of note, *OsSUB16*, *OsSUB18*, *OsSUB23*, *OsSUB32*, *OsSUB36*, *OsSUB35*, *OsSUB39*, and *OsSUB44* were not expressed in any tissues or organs. All of these eight genes belonged to the two largest branches, SUB1 and SUB3, which were subject to purifying selection. This suggests that some genes in clades 1 and 3 exhibit the functional redundancy associated with tandem duplication (*OsSUB16* and *OsSUB44*). *OsSUB5*, *OsSUB7*, *OsSUB9*, *OsSUB16*, *OsSUB13*, *OsSUB14*, *OsSUB15*, and *OsSUB17* were highly expressed in shoots, suggesting that these genes may participate in the development of shoots. Furthermore, we observed that leaves at 20 days displayed the highest expression of *OsSUB21*. More interestingly, four *OsSUBs*, *OsSUB29*, *OsSUB53*, *OsSUB58*, and *OsSUB63,* were specifically expressed during the early stage of seed development, and their expression in seeds at 5 DAP (days after pollination) was higher than that at 10 DAP (Figure 5A). This result encouraged us to explore the role of *OsSUBs* in rice caryopsis development. *OsSUB58* is a member of the SUB3 subfamily, while *OsSUB29*, *OsSUB53*, *and OsSUB63* are classified into the SUB1 clade. In brief, we found that *OsSUBs* exhibit specific expression patterns in different rice tissues.

### *OsSUB29*, *OsSUB53*, *OsSUB58*, and *OsSUB63* Expressed in CPB and CSB May Function in the Early Development of Rice Caryopsis

To gain a preliminary understanding of *OsSUBs* in rice caryopsis development, we targeted four genes (*OsSUB29*, *OsSUB53*, *OsSUB58*, and *OsSUB63*) with higher expression at 5 DAP and measured their expression in CPBs and CSBs at 0, 3, 5, and 12 days after flowering (DAF) using qRT–PCR (Figures 5B–F). In a recent work, the *OsASP1* gene (*aspartic protease 1*, *Os11g08200*) was found to be involved in maintaining the developmental differences of different caryopses on different branches (Chang et al., 2020). Therefore, we considered *OsASP1* a reference gene, and its expression pattern was used to compare the expression pattern of *OsSUBs*. *OsSUB29*, *OsSUB53*, *OsSUB58*, and *OsSUB63* were expressed in caryopses at all developmental stages. The expression patterns of *OsSUB53* and *OsSUB58* were similar to those of *OsASP1*. Except for *OsSUB53*, which was highly expressed in 3-DAF CPB, the relative expression in other types of caryopses was low, even though *OsSUB53* expression in CSB-5 was the second highest (Figure 5C). The highest expression of *OsSUB58* was present in 5 DAF CPB, but the expression was one-seventh of the maximum of *OsSUB53* (Figure 5D). In addition, *OsASP1* expression was highest in 3 DAF CPB, and 5 DAF CSB was the second most *ASP1*-expressing caryopsis type (Figure 5F). *OsSUB53*, *OsSUB58* and *OsASP1* all tended to be highly expressed in early CPBs. However, the expression patterns of *OsSUB29* and *OsSUB63* were significantly different from those of the above three genes, and their relative expression peaks occurred in CSBs, which may be due to gene functional diversity (Figures 5B,E). Expression patterns and levels of *OsSUB53* in caryopses at different developmental stages and positions were most similar to those of *OsASP1*, so *OsSUB53* might share the same signaling pathway with *OsASP1*. In conclusion, these results suggest that *OsSUB29*, *OsSUB53*, *OsSUB58*, and *OsSUB63* are most likely involved in the early development of rice caryopses.

### *OsSUB29*, *OsSUB53*, *OsSUB58*, and *OsSUB63* Have Orthologous Genes From Ancestral *Oryza* Species

**Figure 6:**
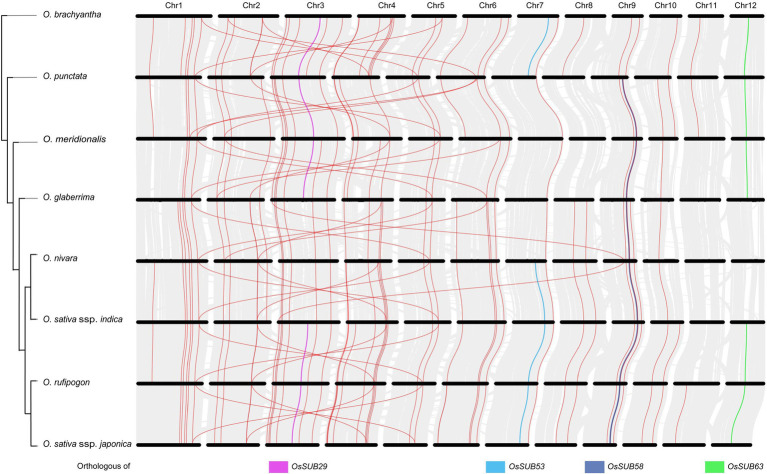
Synteny history of the *SUB* gene family and genomes of *Oryza*. The *Oryza* phylogenetic profile was combined with a synteny relationship map of the whole genome of eight *Oryza* species. Synteny relationships between the genomes of two neighboring species are depicted in gray, and magenta indicates the orthologous gene pairs. The orthologous genes to four genes (*OsSUBs29*, *OsSUBs53*, *OsSUBs58*, *OsSUB63*) are presented in pink, blue, yellow and green, respectively.

To explore the evolutionary history of the subtilisin gene family in depth, we integrated the synteny relationship of *SUBs* among eight *Oryza* species (Figure 6). The genomes between two neighboring species were compared, and the clusters of gray lines represent the orthologues of genes on 12 chromosomes. Seventeen *SUB*s with orthologous relationships were identified across wild and cultivated species, indicating a potential conservation pattern. In combination with the *Oryza* species tree, more orthologous gene pairs were observed among species that were more closely evolutionarily related. The most orthologous gene pairs (48) occurred between *O. rufipogon* and *O. sativa* ssp. *japonica*. Indeed, the crossover of orthologous gene pairs implied that there must be a structural association between chromosomes 2, 4, and 6 in *Oryza* and between chromosomes 1 and 5.

**Figure 7:**
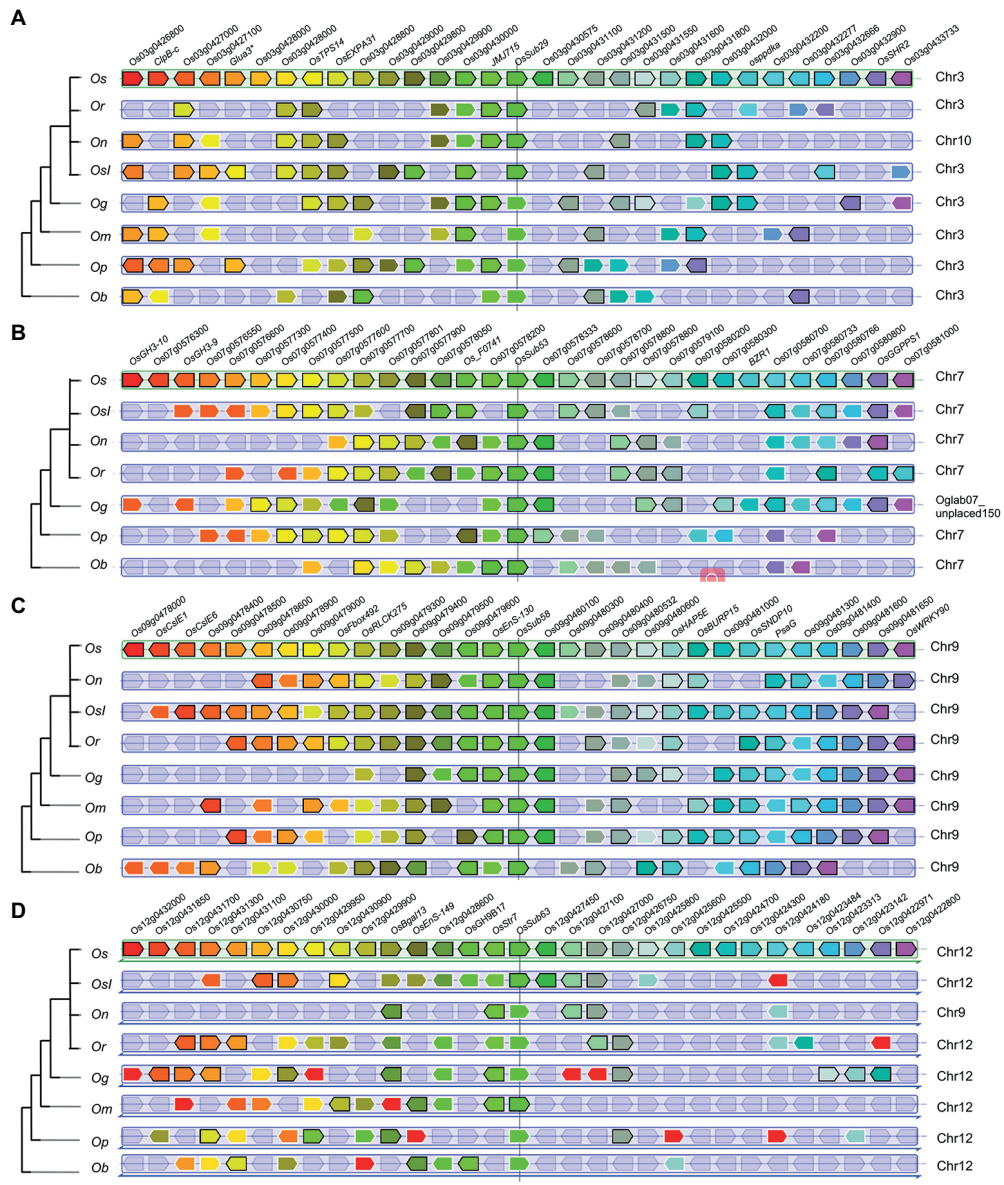
PhyloView of several *OsSUB* reference genes in the rice genome. Phylogenetic species tree of the reference gene is shown on the left. On the right, the reference genes and their homologous copies in other *Oryza* plants are in the center surrounded by their neighboring genes. Homologue genes are colored using the same color. **A–D** Show the PhyloViews of the four genes *OsSUBs29*, *OsSUBs53*, *OsSUBs58*, and *OsSUB63*, which were identified as the reference genes in *O. sativa* ssp. *japonica*, respectively.

Moreover, *OsSUB29*, *OsSUB53*, *OsSUB58*, and *OsSUB63* were highly expressed in early-development rice caryopses and matched their orthologous genes in ancestral wild species (Figure 7). For example, genes orthologous to *OsSUBs53* existed in *O. brachyantha* and *O. punctata* (Ob07G25630, Op07G18770), but a similar situation did not occur in *O. glaberrima* or *O. meridionalis* (Figure 6). Genes with orthologous relationships to *OsSUB53* were again observed among *O. rufipogon*, *O. nivara*, *O. sativa* ssp. *indica*, and *O. sativa* ssp. *japonica* (On07G26510, Ob026418, Or07G20610). However, Figure 7B illustrates that *OsSUB53* possessed an orthologous gene in *O. glaberrima* (Og07G0255300). This was caused by the fact that Og07G0255300 was not precisely annotated on the chromosome. The homologue of *OsSUB53* in *O. meridionalis* may have been lost due to a poor and dramatic environment. Converging our attention on the vicinity of *SUB*s, we found some genes possessing orthologues or paralogues in *Oryza*, either in cultivars or wild species. Figure 7B depicts 30 genes near homologues (or paralogues) in seven *Oryza* plants using *OsSUB53* as the reference gene. *OsGGPPS1*, Os07g0577500, had homologues in seven *Oryza* species, and this conserved state of inheriting a large number of ancestral genes was more obvious, as shown in Figure 7C. However, few direct ancestral homologues were found near the direct homologues of *OsSub64*, which may be related to the dynamic changes in chromosome structure. In addition, several new genes were added near *OsSUB53*, such as *OsGH3-10*, *OsGH3-9*, and Os07g0580800. This suggests that *OsSUB53* had a chance to form a gene cluster with surrounding genes and then play new functions or enhance the original ability.

## Discussion

### The *SUB* Gene Family in *Oryza* Is Updated and Complemented, Including Member Information and Phylogenetic Relationships Among Them

Among serine peptidases, *SUBs* have evolved more diverse functions in plants (Schaller et al., 2018). Currently, many *SUBs* have been characterized in plants, such as *A. thaliana*, *V. vinifera*, *P. trichocarpa*, tomato, and rice (Figueiredo et al., 2018). In 2006, the *SUB* gene family was first explored from the rice genome. As the genome was gradually deep sequenced, the rice genome annotation information became more detailed (Wing et al., 2018). Moreover, transcriptome analysis in rice CPB and CSB reported that *OsSUB53* reached earlier and higher transcriptional peaks in CPB than in CSB after pollination (Chang et al., 2020). Therefore, it is necessary to thoroughly reidentify the *SUBs* from *O. sativa* ssp. *japonica* after 16 years. First, with the help of the annotated whole genome, we considered the presence of 62 *SUB* gene members in *O. sativa* ssp. *japonica*, which is slightly different from that reported in 2006.

Currently, the genus *Oryza* consists of 27 species and is divided into 11 different genomic forms (Kong et al., 2019). The domestication of wild rice and the origin of cultivated rice were also explored. For the first time, across cultivated and wild rice, we extended investigation of the *SUB* gene family to seven other diploid species of *Oryza*. Turning our attention to *O. sativa* ssp. *japonica*, the N-J tree constructed using subtilisin domains described the existence of five major evolutionary branches of *SUBs* (Tripathi and Sowdhamini, 2006). We classified the *SUBs* of *Oryza* into six taxa, and the additional SUB6 branch shared homology with mammalian site-1-protease (S1P) and tripeptidyl peptidase II (TPP2; Schaller et al., 2018). In addition, we found the presence of *SUB* genes from *A. thaliana* and *Oryza* in SUB4. However, it was previously thought that Clade V (SUB4) did not contain the *SUB* genes from rice (Tripathi and Sowdhamini, 2006). If the number of genes in a cluster is too small, it can cause the cluster to be inconspicuous. The phylogenetic analysis containing eight *Oryza* species comprised the SUB4 branch in *Oryza*. The above results facilitated us in further understanding the phylogenetic relationships of *SUBs*. The presence of collinear gene pairs (paralogous homologues) implies a rearrangement or other event in the chromosomes of a species. Two genes on the 2 chromosomes of *O. sativa* ssp. *japonica* constituted collinearity with *SUBs* on chromosomes 4 and 6, respectively. Rice chromosomes 4 and 6 crossover near their telomeres and then lose a tiny part to produce chromosome 2 (Wang et al., 2016). Thus, *SUBs* also provide a molecular example for the evolution of rice chromosomes.

### The Expansion of *SUBs* Is Associated With Tandem Duplication Events and Purifying Selection and May Be the Result of the Continuous Domestication of *Oryza*

Based on the number of *SUBs* in each species, Asian cultivated rice contained more *SUBs* than wild species. To determine the reasons for the above phenomenon, we examined tandem duplication and adaptive selection in a comprehensive manner. Gene family amplification is primarily associated with tandem duplication (TD), segmental duplication (SD), and whole-genome duplication (WGD; Freeling, 2009). In *O. sativa* ssp. *japonica*, we discovered six sets of tandem duplicated gene pairs from 62 *OsSUBs*, and tandem duplication events of *SUBs* were observed in all eight diploid species from *Oryza*. Although TD gene pairs were low in *O. meridionalis*, *O. punctata*, and *O. brachyantha*, the most significant number of TD genes was still maintained in cultivated rice *O. sativa* ssp. *japonica*. Indeed, in *S. tuberosum* and *V. vinifera*, tandem gene duplications are the primary driver of the evolution and expansion of *SUBs* (Cao et al., 2014; Norero et al., 2016). The emergence and extinction of plant gene families can explain species adaptation and lineage evolution (Mitreva et al., 2011). We noted that TD events only occur in clusters 1 and 3 regardless of the species and that TD *SUBs* in SUB3 were subject to more substantial purifying selection. In addition to TD genes being subject to purifying selection, the Overall Average or Sequence Pairs surveys all supported the conclusion that the *SUB* gene family in *Oryza* as a whole underwent purifying selection. Purifying selection is the selective removal of deleterious alleles. It stabilizes selection by purging deleterious genetic polymorphisms that arise through random mutations (Gulisija and Crow, 2007). The expansion of SUB1 and SUB3 was stable and did not evolve rapidly in the recent past, so the genes SUB1 and SUB3 may accumulate more conserved functions. *SUBs* that are increasing in number and undergo purifying selection are highly likely to amplify and intensify their original functions. During rice domestication, populations with domesticated traits (e.g., a higher number of caryopses and a higher filling rate) are retained by cultivators. The molecular mechanisms of shattering four and PROSTRATE GROWTH 1 as standing domestication genes have been continuously resolved by affecting plant architecture or reducing shattering (Li et al., 2006; Jin et al., 2008). *SUBs* probably play an important role in the development of rice caryopses and drive the yield improvement of rice together with domesticated genes. Therefore, the expansion of populations possessing more SUBs in cultivated rice may be the result of rice domestication.

### Four *OsSUBs* Specifically Expressed in Rice Caryopses Have an Earlier Origin in *Oryza*, and the Gene Cluster Formed by *OsSUBs* Together With the Surrounding Gene Blocks May Be Responsible for the Specific Expression of *OsSUBs* in Caryopses

We also focused on the status of *OsSUBs* in different tissues and developmental periods of rice. We found that *OsSUBs* were expressed in a tissue-specific pattern, which implied that *OsSUBs* possessed potential roles in the development of rice. Furthermore, we found that *OsSUB29*, *OsSUB53*, *OsSUB58*, and *OsSUB63* were likely to be involved in the early development of rice caryopses by qRT–PCR. Among them, *OsSUB53* possessed the most similar expression pattern to *OsASP1*, and their transcriptional peaks both occurred at 3 DAF CPB (the relative expression of *OsSUB53* was almost 2-fold higher than that of *OsASP1*).

Figure 7 provides sufficient evidence that *OsSUB29*, *OsSUB53*, *OsSUB58*, and *OsSUB63* existed along with the evolutionary history of *Oryza* rather than being newly generated along with species divergence. In the more domesticated *Oryza*, we also found multiple genes gained in the blocks around the above four *OsSUBs*. Interestingly, recent studies have shown that a single core gene and its nearby completely different genes constitute the gene cluster, which plays an essential role in the metabolic response to plant adversity (Fan et al., 2020). In the vicinity of *OsSUB53*, we observed the appearance of *OsGH3* family members, which modulated both endogenous free IAA and ABA homeostasis (Kong et al., 2019). For water deficit and hypoxic stress, *OsPPDK* was induced to express and newly added around *OsSUB29* (Wang et al., 2018). The gene addition and deletion events were more pronounced around *OsSUB63*, and frequent reversal occurred. *OsGGPPS1* encodes a precursor for synthesizing multiple metabolites required for chloroplast formation (Zhou et al., 2017). Combining the above individual examples, we can deduce that *OsSUBs* were coconstructed with neighboring genes to potentially form a gene cluster. Synteny analysis suggested that the *SUBs*, which were expressed in the rice caryopsis during the early development stage, did not originate during the domestication of *Oryza* species but most likely at the beginning of the independent divergence of the genus *Oryza*. In brief, the gene cluster formed by *OsSUBs* together with the surrounding gene blocks is likely to be responsible for the unique expression of *OsSUBs* in rice caryopsis.

### Summary and Outlook of *SUBs* Related to the Rice Caryopsis

In recent years, we are interested in the problem of rice caryopsis development (Chang et al., 2020). Using a combination of bioinformatics, comparative genomics, and molecular biology approaches, we hope to explore the evolutionary history of the *SUB* gene family in rice, the drivers of expansion, and the relationship with rice caryopsis. Tandem duplication and purifying selection are potential mechanisms for the expansion of *SUBs* in *Oryza*, and more family members appear in more domesticated cultivated rice. This suggests that SUBs with more members may play a role in improving rice yield and caryopsis quality. We show that the specific expression of *OsSUB29*, *OsSUB53*, *OsSUB58*, and *OsSUB63* in the caryopsis is not due to tandem duplication and that their orthologues were found in early wild rice. It cannot be ignored that along with the continuous evolution and domestication of *Oryza* plants, the above four genes formed gene clusters with the surrounding genes. This provides the possibility for the caryopsis quality and phenotype of cultivated rice and explains the specific expression patterns of *OsSUB29*, *OsSUB53*, *OsSUB58*, and *OsSUB63* in CPB and CSB. In short, combining the above results and analysis, we speculate that *OsSUBs* must be involved in the early development of rice caryopsis. However, how *OsSUBs* finely regulate and maintain the differences between CPB and CSB needs further in-depth investigation.

Establishing mutant plants with *OsSUBs*, focusing on the linkage between IAA and *SUBs*, coexpressing gene clusters related to *OsSUBs*, etc., are our next steps to explore the role of *SUBs* in rice caryopsis development. In addition to using cultivated rice as experimental material, collecting a wide variety of wild rice from around the world and conducting comparative studies will surely facilitate the study of caryopsis development.

## Conclusion

Because rice genomic resources are continuously updated and available, we reidentified the presence status of the *SUB* gene family in *O. sativa* ssp. *japonica* after 16 years and seven diploid species from *Oryza* were included. The expansion of *SUBs* in cultivated rice is primarily associated with the occurrence of tandem duplication events and purifying selection and may be the result of rice domestication. Combining the expression patterns of *OsSUBs* in different rice tissues and qRT–PCR results, four *OsSUBs* were expressed in CPB and CSB. *OsSUBs* expressed in rice caryopses have an earlier origin in *Oryza*, and the gene cluster formed by *OsSUBs* together with the surrounding gene blocks may be responsible for the specific expression of *OsSUBs* in caryopses. All the above conclusions are inseparable from the continuous evolution and domestication of *Oryza*.

## Data Availability Statement

The original contributions presented in the study are included in the article/**Supplementary Material**; further inquiries can be directed to the corresponding authors.

## Author Contributions

KZ helped in conceptualization, data curation, investigation, methodology, and writing—original manuscript. LP, XX, and PG helped in investigation and methodology. HZ helped in resources. YW helped in funding acquisition, supervision, and writing—review and editing. SH helped in conceptualization, project administration, supervision, and writing—review and editing. All authors contributed to the article and approved the submitted version.

## Funding

This work was supported by the National Natural Science Foundation of China (grant nos. 30570148 and 31370307). The funders had no role in the study design, data collection and analysis, decision to publish, or preparation of the manuscript.

## Conflict of Interest

The authors declare that the research was conducted in the absence of any commercial or financial relationships that could be construed as a potential conflict of interest.

## Publisher’s Note

All claims expressed in this article are solely those of the authors and do not necessarily represent those of their affiliated organizations, or those of the publisher, the editors and the reviewers. Any product that may be evaluated in this article, or claim that may be made by its manufacturer, is not guaranteed or endorsed by the publisher.

## Footnotes

^1^ http://www.tbtools.org/

^2^ https://blast.ncbi.nlm.nih.gov/Blast.cgi

^3^ https://www.ncbi.nlm.nih.gov/Structure/bwrpsb/bwrpsb.cgi

^4^ https://mafft.cbrc.jp/alignment/server/

^5^ https://www.megasoftware.net/

^6^ http://tree.bio.ed.ac.uk/

^7^ http://chibba.pgml.uga.edu/mcscan2/

^8^ https://www.genomicus.bio.ens.psl.eu/genomicus-plants-49.01/cgi-bin/search.pl
