# Supplementary material for: Genome-Wide Comprehensive Survey of the Subtilisin-Like Proteases Gene Family Associated With Rice Caryopsis Development
Source: Front Plant Sci. 2022 Jun 20;13:943184. doi: 10.3389/fpls.2022.943184 (PMC9251471; doi:10.3389/fpls.2022.943184)
Supplement: Supplementary file 1 [file Table_1.DOCX]

**Supplementary Information**

**Supplementary Table 1. Identification survey of SUB family members in eight *Oryza* species.**

**Supplementary Table 2. General information and sequence characterization of 477 *SUB* genes and protein features from eight *Oryza*.**

**Supplementary Table 3. Primer and probe sequences used in the study (F, forward primer; R, reverse primer).**

**Supplementary Table 4. Update ID of subtilisin gene family members in *O. sativa* ssp. *japonica.***

**Supplementary Table 5. *OsSUB* expression patterns in different rice tissues from the RNA-seq library.**

**Supplementary Table 1. Identification survey of SUB family members in eight *Oryza* species.**

| **Species Name** | **Assembly** | **Accession** | **Number of genes** | **Web** | |  |
| --- | --- | --- | --- | --- | --- | --- |
| *Oryza brachyantha* (*Ob*) | Oryza_brachyantha_v1.4b | GCA_000231095.2 | 45 | http://plants.ensembl.org/index.html | |  |
| *Oryza punctata* (*Op*) | Oryza_punctata_v1.2 | GCA_000573905.1 | 53 |  |  |  |
| *Oryza meridionalis* (*Om*) | Oryza_meridionalis_v1.3 | GCA_000338895.2 | 46 |  |  |  |
| *Oryza glaberrima* (*Og*) | Oryza_glaberrima_V1 | GCA_000147395.1 | 51 |  |  |  |
| *Oryza nivara* (*On*) | Oryza_nivara_v1.0 | GCA_000576065.1 | 52 |  |  |  |
| *O. sativa* ssp. *indica* (*OsI*) | ASM465v1 | GCA_000004655.2 | 61 |  |  |  |
| *Oryza rufipogon* (*Or*) | OR_W1943 | GCA_000817225.1 | 52 | |  | |
| *O. sativa ssp. japonica* (*Os*) | IRGSP-1.0 | GCA_001433935.1 | 62 | http://rice.uga.edu/index.shtml | |  |
| *Arabidopsis thaliana* (*At*) | TAIR10 | GCA_000001735.1 | 56 (55) | http://plants.ensembl.org/index.html | |  |

**Supplementary Table 2. General information and sequence characterization of 477 *SUB* genes and protein features from eight *Oryza*.**

| Gene Name | Sequence ID | MW (Da) | pI |
| --- | --- | --- | --- |
| Ob04G28450 | OB04G28450 | 70121.29 | 5.80 |
| Ob02G16820 | OB02G16820 | 74044.73 | 9.29 |
| Ob04G10860 | OB04G10860 | 80496.50 | 7.28 |
| Ob07G28330 | OB07G28330 | 73888.33 | 7.01 |
| Ob07G25640 | OB07G25640 | 80288.86 | 8.65 |
| Ob03G42030 | OB03G42030 | 76685.00 | 6.80 |
| Ob06G23090 | OB06G23090 | 72557.36 | 6.02 |
| Ob10G23440 | OB10G23440 | 79094.10 | 5.33 |
| Ob03G33800 | OB03G33800 | 51662.28 | 5.87 |
| Ob04G27390 | OB04G27390 | 72086.82 | 6.18 |
| Ob04G28440 | OB04G28440 | 80463.40 | 6.66 |
| Ob02G20360 | OB02G20360 | 85674.37 | 6.39 |
| Ob03G11460 | OB03G11460 | 77975.56 | 6.41 |
| Ob07G25630 | OB07G25630 | 90989.58 | 8.31 |
| Ob08G19420 | OB08G19420 | 82975.79 | 6.17 |
| Ob03G31300 | OB03G31300 | 71727.31 | 7.96 |
| Ob06G13940 | OB06G13940 | 97901.07 | 5.73 |
| Ob01G42610 | OB01G42610 | 79578.15 | 7.64 |
| Ob07G18030 | OB07G18030 | 82988.04 | 6.68 |
| Ob01G41240 | OB01G41240 | 88971.62 | 6.14 |
| Ob01G37010 | OB01G37010 | 75378.16 | 5.56 |
| Ob04G29350 | OB04G29350 | 80347.84 | 6.45 |
| Ob09G23970 | OB09G23970 | 82153.54 | 8.59 |
| Ob04G10970 | OB04G10970 | 80750.69 | 6.12 |
| Ob01G42580 | OB01G42580 | 79925.27 | 6.56 |
| Ob04G11020 | OB04G11020 | 79828.78 | 8.32 |
| Ob04G20110 | OB04G20110 | 80834.40 | 8.98 |
| Ob05G21890 | OB05G21890 | 69932.12 | 6.71 |
| Ob06G33960 | OB06G33960 | 87086.16 | 6.52 |
| Ob03G20330 | OB03G20330 | 76553.70 | 8.23 |
| Ob12G18890 | OB12G18890 | 78964.59 | 6.07 |
| Ob06G29360 | OB06G29360 | 53739.64 | 8.45 |
| Ob05G25620 | OB05G25620 | 79012.53 | 6.68 |
| Ob02G34290 | OB02G34290 | 84013.04 | 6.54 |
| Ob11G17030 | OB11G17030 | 81140.66 | 6.17 |
| Ob01G42570 | OB01G42570 | 79347.50 | 6.59 |
| Ob04G10880 | OB04G10880 | 80244.22 | 6.50 |
| Ob02G20410 | OB02G20410 | 87271.84 | 7.97 |
| Ob07G32290 | OB07G32290 | 78504.59 | 6.12 |
| Ob09G18290 | OB09G18290 | 79385.31 | 8.37 |
| Ob01G21410 | OB01G21410 | 119018.08 | 8.68 |
| Ob02G34230 | OB02G34230 | 148907.46 | 5.87 |
| Ob03G13380 | OB03G13380 | 81227.39 | 8.32 |
| Ob01G47320 | OB01G47320 | 68568.09 | 7.43 |
| Ob09G20640 | OB09G20640 | 79083.80 | 9.54 |
| Og04G0009000 | ORGLA04G0009000 | 81257.66 | 6.39 |
| Og02G0300100 | ORGLA02G0300100 | 76131.71 | 7.00 |
| Og08G0094200 | ORGLA08G0094200 | 81793.70 | 6.17 |
| Og08G0147100 | ORGLA08G0147100 | 81362.29 | 8.54 |
| Og02G0232800 | ORGLA02G0232800 | 84106.02 | 8.14 |
| Og04G0099500 | ORGLA04G0099500 | 80598.58 | 8.03 |
| Og09G0109700 | ORGLA09G0109700 | 82736.96 | 8.32 |
| Og02G0300200 | ORGLA02G0300200 | 80955.26 | 5.89 |
| Og04G0184600 | ORGLA04G0184600 | 83691.52 | 7.57 |
| Og09G0171000 | ORGLA09G0171000 | 81362.29 | 8.54 |
| Og01G0264100 | ORGLA01G0264100 | 90122.89 | 6.17 |
| Og05G0119200 | ORGLA05G0119200 | 80553.77 | 6.04 |
| Og06G0177000 | ORGLA06G0177000 | 94252.03 | 9.11 |
| Og09G0088700 | ORGLA09G0088700 | 79577.88 | 7.31 |
| Og02G0105500 | ORGLA02G0105500 | 78367.18 | 6.65 |
| Og07G0211900 | ORGLA07G0211900 | 69637.79 | 5.65 |
| Og04G0193700 | ORGLA04G0193700 | 82110.01 | 6.18 |
| Og06G0219900 | ORGLA06G0219900 | 87577.77 | 7.10 |
| Og01G0277000 | ORGLA01G0277000 | 79868.53 | 7.30 |
| Og02G0105600 | ORGLA02G0105600 | 80756.83 | 8.52 |
| Og03G0207500 | ORGLA03G0207500 | 80671.02 | 6.09 |
| Og01G0243500 | ORGLA01G0243500 | 81193.20 | 7.62 |
| Og02G0232300 | ORGLA02G0232300 | 148696.05 | 6.15 |
| Og03G0097800 | ORGLA03G0097800 | 80072.52 | 7.29 |
| Og04G0009100 | ORGLA04G0009100 | 81485.69 | 6.61 |
| Og09G0135900 | ORGLA09G0135900 | 81989.60 | 8.73 |
| Og06G0039100 | ORGLA06G0039100 | 116488.77 | 6.01 |
| Og03G0031300 | ORGLA03G0031300 | 83067.08 | 8.47 |
| Og01G0276800 | ORGLA01G0276800 | 79083.82 | 6.29 |
| Og01G0324600 | ORGLA01G0324600 | 79150.29 | 5.21 |
| Og02G0105700 | ORGLA02G0105700 | 78674.53 | 7.24 |
| Og03G0320700 | ORGLA03G0320700 | 78529.19 | 6.19 |
| Og06G0171200 | ORGLA06G0171200 | 84176.47 | 7.64 |
| Og02G0105800 | ORGLA02G0105800 | 78818.78 | 6.48 |
| Og04G0008800 | ORGLA04G0008800 | 77291.16 | 7.33 |
| Og01G0324700 | ORGLA01G0324700 | 78699.18 | 8.61 |
| Og01G0276900 | ORGLA01G0276900 | 72865.85 | 6.08 |
| Og09G0107600 | ORGLA09G0107600 | 84601.94 | 9.41 |
| Og04G0022200 | ORGLA04G0022200 | 79384.03 | 8.94 |
| Og02G0105900 | ORGLA02G0105900 | 71745.05 | 7.54 |
| Og03G0237000 | ORGLA03G0237000 | 78175.08 | 5.89 |
| Og04G0010000 | ORGLA04G0010000 | 82071.70 | 6.22 |
| Og02G0070800 | ORGLA02G0070800 | 83224.43 | 8.30 |
| Og03G0012900 | ORGLA03G0012900 | 78945.84 | 6.56 |
| Og04G0184700 | ORGLA04G0184700 | 79317.71 | 6.19 |
| Og10G0074500 | ORGLA10G0074500 | 79127.75 | 9.45 |
| Og05G0153900 | ORGLA05G0153900 | 79623.18 | 6.51 |
| Og02G0301000 | ORGLA02G0301000 | 77119.31 | 8.37 |
| Og03G0041800 | ORGLA03G0041800 | 85442.61 | 6.10 |
| Og12G0095800 | ORGLA12G0095800 | 67621.70 | 5.81 |
| Og01G0229700 | ORGLA01G0229700 | 78015.29 | 5.77 |
| Om02G12340 | OMERI02G12340 | 156187.28 | 7.20 |
| Om01G30640 | OMERI01G30640 | 79658.28 | 7.30 |
| Om09G10850 | OMERI09G10850 | 84759.09 | 9.38 |
| Om10G12890 | OMERI10G12890 | 95577.52 | 6.20 |
| Om03G03330 | OMERI03G03330 | 83082.03 | 8.34 |
| Om04G17870 | OMERI04G17870 | 83543.28 | 7.88 |
| Om04G00980 | OMERI04G00980 | 120048.09 | 7.28 |
| Om03G32250 | OMERI03G32250 | 78660.32 | 6.19 |
| Om01G11300 | OMERI01G11300 | 83103.10 | 8.36 |
| Om01G30590 | OMERI01G30590 | 79205.47 | 5.96 |
| Om03G22590 | OMERI03G22590 | 81519.44 | 8.40 |
| Om04G01000 | OMERI04G01000 | 81710.75 | 6.08 |
| Om07G23400 | OMERI07G23400 | 80968.69 | 6.00 |
| Om02G32890 | OMERI02G32890 | 81893.72 | 6.36 |
| Om01G29320 | OMERI01G29320 | 89285.78 | 6.05 |
| Om04G17880 | OMERI04G17880 | 79861.42 | 6.17 |
| Om08G09010 | OMERI08G09010 | 81715.58 | 6.17 |
| Om10G06030 | OMERI10G06030 | 78781.36 | 9.40 |
| Om05G12330 | OMERI05G12330 | 80410.62 | 5.93 |
| Om01G30610 | OMERI01G30610 | 95254.12 | 5.99 |
| Om06G04900 | OMERI06G04900 | 117896.66 | 6.14 |
| Om09G09240 | OMERI09G09240 | 79809.04 | 6.87 |
| Om04G11570 | OMERI04G11570 | 80218.38 | 8.01 |
| Om01G35530 | OMERI01G35530 | 79005.16 | 5.33 |
| Om09G11090 | OMERI09G11090 | 82525.78 | 6.92 |
| Om06G26510 | OMERI06G26510 | 90463.91 | 6.29 |
| Om04G01090 | OMERI04G01090 | 105340.26 | 6.55 |
| Om03G04150 | OMERI03G04150 | 106606.43 | 9.42 |
| Om01G36990 | OMERI01G36990 | 57878.68 | 7.27 |
| Om11G07870 | OMERI11G07870 | 81413.20 | 7.28 |
| Om03G09430 | OMERI03G09430 | 80178.64 | 7.29 |
| Om01G35540 | OMERI01G35540 | 78785.27 | 8.27 |
| Om03G23410 | OMERI03G23410 | 78136.06 | 5.79 |
| Om02G08590 | OMERI02G08590 | 82447.44 | 8.46 |
| Om02G12330 | OMERI02G12330 | 80482.50 | 7.20 |
| Om01G39840 | OMERI01G39840 | 71993.07 | 4.84 |
| Om02G26220 | OMERI02G26220 | 148708.16 | 6.08 |
| Om03G20100 | OMERI03G20100 | 80574.99 | 6.32 |
| Om12G06960 | OMERI12G06960 | 78833.49 | 6.42 |
| Om01G31200 | OMERI01G31200 | 89172.48 | 8.97 |
| Om02G32900 | OMERI02G32900 | 80949.10 | 5.76 |
| Om05G15410 | OMERI05G15410 | 79524.99 | 6.33 |
| Om06G21250 | OMERI06G21250 | 87950.64 | 6.89 |
| Om04G02940 | OMERI04G02940 | 79625.64 | 9.01 |
| Om02G32870 | OMERI02G32870 | 77768.63 | 8.59 |
| Om03G01490 | OMERI03G01490 | 78983.97 | 6.66 |
| On02G09060 | ONIVA02G09060 | 82476.54 | 8.32 |
| On08G11400 | ONIVA08G11400 | 81809.66 | 6.11 |
| On09G11610 | ONIVA09G11610 | 79328.25 | 6.47 |
| On01G16720 | ONIVA01G16720 | 79228.81 | 8.79 |
| On02G15650 | ONIVA02G15650 | 83313.38 | 7.06 |
| On04G00890 | ONIVA04G00890 | 78717.19 | 6.93 |
| On01G36680 | ONIVA01G36680 | 89518.13 | 6.17 |
| On01G33860 | ONIVA01G33860 | 80840.75 | 7.62 |
| On01G38530 | ONIVA01G38530 | 299639.18 | 6.37 |
| On06G28880 | ONIVA06G28880 | 88601.03 | 6.91 |
| On04G19920 | ONIVA04G19920 | 83568.41 | 7.57 |
| On06G24420 | ONIVA06G24420 | 119306.28 | 6.26 |
| On06G25300 | ONIVA06G25300 | 94189.85 | 8.95 |
| On02G29450 | ONIVA02G29450 | 149687.14 | 6.02 |
| On01G38560 | ONIVA01G38560 | 79780.45 | 7.63 |
| On09G15310 | ONIVA09G15310 | 78824.46 | 6.23 |
| On03G36550 | ONIVA03G36550 | 78189.86 | 6.19 |
| On09G18380 | ONIVA09G18380 | 82017.67 | 8.73 |
| On12G14840 | ONIVA12G14840 | 81517.71 | 6.61 |
| On07G26510 | ONIVA07G26510 | 81025.87 | 5.96 |
| On08G18360 | ONIVA08G18360 | 81374.30 | 8.40 |
| On01G43460 | ONIVA01G43460 | 159227.17 | 6.39 |
| On04G00790 | ONIVA04G00790 | 81251.96 | 8.39 |
| On02G13170 | ONIVA02G13170 | 201001.69 | 6.61 |
| On07G18280 | ONIVA07G18280 | 81555.65 | 6.24 |
| On03G04150 | ONIVA03G04150 | 86059.31 | 6.10 |
| On04G19930 | ONIVA04G19930 | 78823.08 | 6.22 |
| On03G10800 | ONIVA03G10800 | 80082.56 | 7.02 |
| On09G12110 | ONIVA09G12110 | 79505.81 | 7.64 |
| On02G36800 | ONIVA02G36800 | 75614.96 | 6.59 |
| On01G13730 | ONIVA01G13730 | 80646.20 | 7.61 |
| On05G18660 | ONIVA05G18660 | 79611.13 | 6.51 |
| On06G05440 | ONIVA06G05440 | 118125.43 | 5.98 |
| On02G36770 | ONIVA02G36770 | 79986.23 | 5.89 |
| On09G14850 | ONIVA09G14850 | 82749.96 | 8.32 |
| On10G14240 | ONIVA10G14240 | 80730.07 | 6.09 |
| On02G13160 | ONIVA02G13160 | 81577.96 | 7.24 |
| On04G00970 | ONIVA04G00970 | 61352.21 | 7.92 |
| On03G26700 | ONIVA03G26700 | 77991.89 | 5.88 |
| On04G00780 | ONIVA04G00780 | 82210.04 | 8.17 |
| On04G11110 | ONIVA04G11110 | 80467.39 | 8.03 |
| On09G14620 | ONIVA09G14620 | 84598.94 | 9.41 |
| On10G08450 | ONIVA10G08450 | 79166.88 | 9.45 |
| On03G03090 | ONIVA03G03090 | 83078.06 | 7.93 |
| On01G31800 | ONIVA01G31800 | 76984.82 | 5.59 |
| On04G20930 | ONIVA04G20930 | 82110.01 | 6.18 |
| On02G36830 | ONIVA02G36830 | 81579.18 | 7.32 |
| On05G14400 | ONIVA05G14400 | 80417.62 | 6.11 |
| On02G36760 | ONIVA02G36760 | 106921.58 | 6.62 |
| On02G29540 | ONIVA02G29540 | 84145.10 | 8.30 |
| On12G00720 | ONIVA12G00720 | 79406.43 | 5.26 |
| On02G13150 | ONIVA02G13150 | 73771.84 | 6.35 |
| Op09G10290 | OPUNC09G10290 | 86901.70 | 5.85 |
| Op03G23610 | OPUNC03G23610 | 77984.87 | 5.71 |
| Op04G01110 | OPUNC04G01110 | 82078.58 | 6.51 |
| Op01G31370 | OPUNC01G31370 | 89437.12 | 6.17 |
| Op11G08480 | OPUNC11G08480 | 96980.15 | 9.11 |
| Op08G09970 | OPUNC08G09970 | 77282.81 | 6.12 |
| Op02G24660 | OPUNC02G24660 | 72947.33 | 8.49 |
| Op05G15700 | OPUNC05G15700 | 72526.36 | 6.15 |
| Op03G01410 | OPUNC03G01410 | 79384.35 | 6.67 |
| Op03G32070 | OPUNC03G32070 | 78938.74 | 6.22 |
| Op08G14720 | OPUNC08G14720 | 81216.04 | 8.19 |
| Op09G12560 | OPUNC09G12560 | 131401.04 | 6.81 |
| Op04G01200 | OPUNC04G01200 | 75273.24 | 5.74 |
| Op05G12010 | OPUNC05G12010 | 80224.62 | 5.89 |
| Op12G09210 | OPUNC12G09210 | 83787.07 | 6.66 |
| Op04G03010 | OPUNC04G03010 | 80031.86 | 9.11 |
| Op04G01140 | OPUNC04G01140 | 74576.47 | 8.45 |
| Op01G37360 | OPUNC01G37360 | 157937.52 | 6.03 |
| Op06G23940 | OPUNC06G23940 | 87300.26 | 6.77 |
| Op06G19440 | OPUNC06G19440 | 88638.96 | 8.43 |
| Op02G10400 | OPUNC02G10400 | 78879.73 | 6.76 |
| Op03G23580 | OPUNC03G23580 | 77984.87 | 5.71 |
| Op02G32020 | OPUNC02G32020 | 77057.95 | 8.45 |
| Op04G18100 | OPUNC04G18100 | 89845.82 | 5.66 |
| Op02G06740 | OPUNC02G06740 | 76945.90 | 7.94 |
| Op03G04070 | OPUNC03G04070 | 86463.21 | 6.91 |
| Op04G10930 | OPUNC04G10930 | 80205.48 | 8.25 |
| Op07G25170 | OPUNC07G25170 | 80582.38 | 6.26 |
| Op04G19100 | OPUNC04G19100 | 79083.56 | 6.04 |
| Op02G10390 | OPUNC02G10390 | 77291.82 | 6.32 |
| Op01G32820 | OPUNC01G32820 | 297536.88 | 7.77 |
| Op09G12800 | OPUNC09G12800 | 99996.32 | 7.84 |
| Op02G24590 | OPUNC02G24590 | 148491.98 | 6.15 |
| Op01G29350 | OPUNC01G29350 | 80732.59 | 6.86 |
| Op04G19090 | OPUNC04G19090 | 83717.60 | 8.12 |
| Op10G07600 | OPUNC10G07600 | 79607.27 | 9.41 |
| Op02G32030 | OPUNC02G32030 | 78111.65 | 6.42 |
| Op02G10410 | OPUNC02G10410 | 78586.20 | 5.68 |
| Op01G27740 | OPUNC01G27740 | 78196.65 | 5.58 |
| Op01G11180 | OPUNC01G11180 | 65525.44 | 9.00 |
| Op10G14790 | OPUNC10G14790 | 79013.00 | 5.17 |
| Op07G18770 | OPUNC07G18770 | 81145.14 | 6.23 |
| Op06G18850 | OPUNC06G18850 | 83281.08 | 6.37 |
| Op01G32850 | OPUNC01G32850 | 79630.34 | 8.12 |
| Op03G20980 | OPUNC03G20980 | 80370.64 | 6.15 |
| Op04G20060 | OPUNC04G20060 | 82085.00 | 6.26 |
| Op09G15740 | OPUNC09G15740 | 86358.58 | 8.48 |
| Op04G01230 | OPUNC04G01230 | 78224.97 | 6.11 |
| Op02G10420 | OPUNC02G10420 | 79381.57 | 6.58 |
| Op06G04060 | OPUNC06G04060 | 116382.68 | 5.91 |
| Op02G32040 | OPUNC02G32040 | 81287.73 | 6.12 |
| Op03G03090 | OPUNC03G03090 | 75025.35 | 8.39 |
| Op03G09780 | OPUNC03G09780 | 80303.91 | 7.65 |
| Or01G35590 | ORUFI01G35590 | 89545.09 | 6.17 |
| Or10G09200 | ORUFI10G09200 | 79149.80 | 9.41 |
| Or06G28270 | ORUFI06G28270 | 88645.08 | 6.91 |
| Or03G01280 | ORUFI03G01280 | 79328.25 | 6.47 |
| Or04G23980 | ORUFI04G23980 | 82110.01 | 6.18 |
| Or04G01450 | ORUFI04G01450 | 77901.56 | 5.63 |
| Or02G36270 | ORUFI02G36270 | 81623.18 | 7.32 |
| Or09G15340 | ORUFI09G15340 | 82778.01 | 8.32 |
| Or02G07640 | ORUFI02G07640 | 83172.37 | 7.89 |
| Or07G20610 | ORUFI07G20610 | 81566.68 | 6.24 |
| Or04G22990 | ORUFI04G22990 | 79328.57 | 6.19 |
| Or04G01160 | ORUFI04G01160 | 147675.11 | 6.33 |
| Or05G14990 | ORUFI05G14990 | 80554.76 | 5.97 |
| Or08G18730 | ORUFI08G18730 | 82298.27 | 8.36 |
| Or04G22030 | ORUFI04G22030 | 89245.30 | 6.27 |
| Or12G11310 | ORUFI12G11310 | 78722.35 | 6.42 |
| Or05G18980 | ORUFI05G18980 | 79581.10 | 6.51 |
| Or03G03090 | ORUFI03G03090 | 83038.98 | 7.64 |
| Or01G12350 | ORUFI01G12350 | 123837.85 | 6.81 |
| Or06G22110 | ORUFI06G22110 | 119322.33 | 6.32 |
| Or03G10380 | ORUFI03G10380 | 80096.63 | 7.29 |
| Or03G36500 | ORUFI03G36500 | 78502.17 | 6.19 |
| Or11G09320 | ORUFI11G09320 | 78109.29 | 6.67 |
| Or07G27730 | ORUFI07G27730 | 81054.87 | 6.05 |
| Or01G42000 | ORUFI01G42000 | 159119.06 | 6.27 |
| Or09G12600 | ORUFI09G12600 | 79531.89 | 7.64 |
| Or03G23000 | ORUFI03G23000 | 78813.03 | 6.50 |
| Or04G22980 | ORUFI04G22980 | 83568.41 | 7.57 |
| Or03G26460 | ORUFI03G26460 | 77991.89 | 5.88 |
| Or04G01130 | ORUFI04G01130 | 138305.39 | 6.81 |
| Or01G37000 | ORUFI01G37000 | 72996.21 | 6.55 |
| Or08G11940 | ORUFI08G11940 | 70976.36 | 5.96 |
| Or02G11790 | ORUFI02G11790 | 199783.19 | 7.68 |
| Or02G28500 | ORUFI02G28500 | 84130.09 | 8.14 |
| Or01G32680 | ORUFI01G32680 | 80853.79 | 7.28 |
| Or04G14110 | ORUFI04G14110 | 80467.39 | 8.03 |
| Or02G36210 | ORUFI02G36210 | 83234.91 | 6.02 |
| Or02G11760 | ORUFI02G11760 | 80889.16 | 7.89 |
| Or02G28400 | ORUFI02G28400 | 148632.99 | 6.15 |
| Or01G36980 | ORUFI01G36980 | 67883.30 | 8.01 |
| Or02G36250 | ORUFI02G36250 | 71686.55 | 6.38 |
| Or06G04250 | ORUFI06G04250 | 118153.49 | 5.98 |
| Or01G37010 | ORUFI01G37010 | 163317.06 | 6.48 |
| Or03G04060 | ORUFI03G04060 | 86089.34 | 6.10 |
| Or06G22970 | ORUFI06G22970 | 93522.16 | 9.07 |
| Or09G18730 | ORUFI09G18730 | 92182.84 | 9.28 |
| Or10G17390 | ORUFI10G17390 | 79221.21 | 5.26 |
| Or09G15100 | ORUFI09G15100 | 84643.00 | 9.41 |
| Or02G11750 | ORUFI02G11750 | 78704.82 | 6.96 |
| Or01G30910 | ORUFI01G30910 | 74421.08 | 5.77 |
| Or04G03720 | ORUFI04G03720 | 79320.93 | 8.94 |
| Or02G36200 | ORUFI02G36200 | 115426.38 | 7.37 |
| OsSUB2 | LOC_Os01g50680 | 44482.16 | 5.73 |
| OsSUB3 | LOC_Os01g52750 | 81128.13 | 7.62 |
| OsSUB4 | LOC_Os01g56320 | 89545.09 | 6.17 |
| OsSUB5 | LOC_Os01g58240 | 78856.36 | 6.10 |
| OsSUB7 | LOC_Os01g58270 | 86099.77 | 7.32 |
| OsSUB8 | LOC_Os01g58280 | 79703.83 | 6.65 |
| OsSUB9 | LOC_Os01g58290 | 79775.43 | 7.63 |
| OsSUB10 | LOC_Os01g64850 | 79112.40 | 5.15 |
| OsSUB11 | LOC_Os01g64860 | 78811.31 | 8.72 |
| OsSUB12 | LOC_Os02g10520 | 83172.37 | 7.89 |
| OsSUB13 | LOC_Os02g16940 | 78093.80 | 6.63 |
| OsSUB14 | LOC_Os02g17000 | 53782.76 | 7.24 |
| OsSUB15 | LOC_Os02g17060 | 60077.40 | 7.11 |
| OsSUB16 | LOC_Os02g17080 | 63586.44 | 8.24 |
| OsSUB17 | LOC_Os02g17090 | 78605.50 | 6.96 |
| OsSUB18 | LOC_Os02g17150 | 71700.93 | 7.54 |
| OsSUB19 | LOC_Os02g44520 | 148632.99 | 6.15 |
| OsSUB20 | LOC_Os02g44590 | 84201.21 | 8.30 |
| OsSUB21 | LOC_Os02g53850 | 76219.72 | 7.29 |
| OsSUB22 | LOC_Os02g53860 | 80840.11 | 5.95 |
| OsSUB23 | LOC_Os02g53910 | 72825.77 | 6.52 |
| OsSUB24 | LOC_Os02g53970 | 83600.59 | 7.35 |
| OsSUB25 | LOC_Os03g02750 | 79372.26 | 6.39 |
| OsSUB26 | LOC_Os03g04950 | 83038.98 | 7.64 |
| OsSUB27 | LOC_Os03g06290 | 85287.41 | 6.10 |
| OsSUB28 | LOC_Os03g13930 | 80096.63 | 7.29 |
| OsSUB29 | LOC_Os03g31630 | 80743.22 | 6.19 |
| OsSUB30 | LOC_Os03g40830 | 77991.89 | 5.88 |
| OsSUB31 | LOC_Os03g55350 | 78502.17 | 6.19 |
| OsSUB32 | LOC_Os04g02960 | 304255.73 | 8.22 |
| OsSUB33 | LOC_Os04g02980 | 74257.77 | 8.09 |
| OsSUB34 | LOC_Os04g03050 | 81249.72 | 6.50 |
| OsSUB35 | LOC_Os04g03100 | 81785.97 | 6.15 |
| OsSUB36 | LOC_Os04g03710 | 137046.89 | 6.44 |
| OsSUB37 | LOC_Os04g03796 | 83068.63 | 5.58 |
| OsSUB38 | LOC_Os04g03810 | 79721.98 | 7.32 |
| OsSUB39 | LOC_Os04g03850 | 50854.61 | 7.93 |
| OsSUB40 | LOC_Os04g10360 | 79399.14 | 8.94 |
| OsSUB41 | LOC_Os04g35140 | 80598.58 | 8.03 |
| OsSUB42 | LOC_Os04g45960 | 88644.84 | 6.26 |
| OsSUB43 | LOC_Os04g47150 | 83568.41 | 7.57 |
| OsSUB44 | LOC_Os04g47160 | 79387.72 | 6.19 |
| OsSUB45 | LOC_Os04g48416 | 82140.04 | 6.18 |
| OsSUB46 | LOC_Os05g30580 | 80554.76 | 5.97 |
| OsSUB47 | LOC_Os05g36010 | 79581.10 | 6.51 |
| OsSUB48 | LOC_Os06g06802 | 209148.97 | 8.22 |
| OsSUB49 | LOC_Os06g06810 | 75929.35 | 6.35 |
| OsSUB50 | LOC_Os06g40700 | 83542.65 | 6.61 |
| OsSUB51 | LOC_Os06g41880 | 109924.96 | 9.33 |
| OsSUB52 | LOC_Os06g48650 | 87423.55 | 6.77 |
| OsSUB53 | LOC_Os07g39020 | 81566.68 | 6.24 |
| OsSUB54 | LOC_Os07g48650 | 81287.22 | 5.96 |
| OsSUB55 | LOC_Os08g23740 | 81809.66 | 6.11 |
| OsSUB56 | LOC_Os08g35090 | 81360.27 | 8.40 |
| OsSUB57 | LOC_Os09g26920 | 79505.81 | 7.64 |
| OsSUB58 | LOC_Os09g30250 | 84633.94 | 9.37 |
| OsSUB59 | LOC_Os09g36110 | 81988.61 | 8.73 |
| OsSUB60 | LOC_Os10g25450 | 79132.80 | 9.45 |
| OsSUB61 | LOC_Os10g38080 | 79467.49 | 5.17 |
| OsSUB62 | LOC_Os11g15520 | 79872.77 | 8.61 |
| OsSUB63 | LOC_Os12g23980 | 78813.43 | 6.23 |
| OsSUB64 | LOC_Os09g30458 | 82758.97 | 8.15 |
| OsI016862 | BGIOSGA016862 | 93389.26 | 6.37 |
| OsI006725 | BGIOSGA006725 | 78093.80 | 6.63 |
| OsI004601 | BGIOSGA004601 | 84169.22 | 6.91 |
| OsI006721 | BGIOSGA006721 | 71700.93 | 7.54 |
| OsI016910 | BGIOSGA016910 | 61038.15 | 8.89 |
| OsI016436 | BGIOSGA016436 | 59456.04 | 8.89 |
| OsI004599 | BGIOSGA004599 | 151990.11 | 6.66 |
| OsI005492 | BGIOSGA005492 | 55369.23 | 5.50 |
| OsI000483 | BGIOSGA000483 | 84022.85 | 5.09 |
| OsI030974 | BGIOSGA030974 | 126804.24 | 8.59 |
| OsI013093 | BGIOSGA013093 | 47389.45 | 6.99 |
| OsI030529 | BGIOSGA030529 | 69828.32 | 5.72 |
| OsI014520 | BGIOSGA014520 | 48684.61 | 9.00 |
| OsI029566 | BGIOSGA029566 | 82763.00 | 8.32 |
| OsI004600 | BGIOSGA004600 | 87209.01 | 7.32 |
| OsI014562 | BGIOSGA014562 | 79415.78 | 6.19 |
| OsI006722 | BGIOSGA006722 | 78605.50 | 6.96 |
| OsI011416 | BGIOSGA011416 | 74104.31 | 5.85 |
| OsI012929 | BGIOSGA012929 | 86412.47 | 7.32 |
| OsI006938 | BGIOSGA006938 | 82428.45 | 8.15 |
| OsI023250 | BGIOSGA023250 | 119933.08 | 6.16 |
| OsI008770 | BGIOSGA008770 | 84145.10 | 8.30 |
| OsI011836 | BGIOSGA011836 | 84429.62 | 8.31 |
| OsI028453 | BGIOSGA028453 | 43306.76 | 5.57 |
| OsI032858 | BGIOSGA032858 | 79140.80 | 9.45 |
| OsI005488 | BGIOSGA005488 | 81579.18 | 7.32 |
| OsI026007 | BGIOSGA026007 | 81539.66 | 6.24 |
| OsI009771 | BGIOSGA009771 | 46422.35 | 7.65 |
| OsI011133 | BGIOSGA011133 | 57427.16 | 6.53 |
| OsI011567 | BGIOSGA011567 | 56269.94 | 7.24 |
| OsI017986 | BGIOSGA017986 | 58800.85 | 8.01 |
| OsI015801 | BGIOSGA015801 | 55942.03 | 8.75 |
| OsI008767 | BGIOSGA008767 | 148666.06 | 6.15 |
| OsI006723 | BGIOSGA006723 | 89227.84 | 8.51 |
| OsI015799 | BGIOSGA015799 | 36038.12 | 4.69 |
| OsI031138 | BGIOSGA031138 | 82017.67 | 8.73 |
| OsI036297 | BGIOSGA036297 | 73127.92 | 6.33 |
| OsI019733 | BGIOSGA019733 | 80516.67 | 6.04 |
| OsI028455 | BGIOSGA028455 | 136595.39 | 6.21 |
| OsI015814 | BGIOSGA015814 | 79650.38 | 5.88 |
| OsI004876 | BGIOSGA004876 | 57497.08 | 4.98 |
| OsI030324 | BGIOSGA030324 | 56477.27 | 9.11 |
| OsI003258 | BGIOSGA003258 | 80371.92 | 7.61 |
| OsI030840 | BGIOSGA030840 | 79505.81 | 7.64 |
| OsI023552 | BGIOSGA023552 | 94542.29 | 8.93 |
| OsI004526 | BGIOSGA004526 | 89418.99 | 6.09 |
| OsI031523 | BGIOSGA031523 | 79406.43 | 5.26 |
| OsI006724 | BGIOSGA006724 | 84297.75 | 7.25 |
| OsI026418 | BGIOSGA026418 | 80967.83 | 6.04 |
| OsI023290 | BGIOSGA023290 | 86120.92 | 9.15 |
| OsI000886 | BGIOSGA000886 | 80826.73 | 7.62 |
| OsI035372 | BGIOSGA035372 | 83080.26 | 7.88 |
| OsI022373 | BGIOSGA022373 | 101930.17 | 5.86 |
| OsI009142 | BGIOSGA009142 | 76526.22 | 7.26 |
| OsI015817 | BGIOSGA015817 | 61305.21 | 8.19 |
| OsI028775 | BGIOSGA028775 | 72140.84 | 8.67 |
| OsI004877 | BGIOSGA004877 | 57918.06 | 8.89 |
| OsI004277 | BGIOSGA004277 | 77972.24 | 5.50 |
| OsI005493 | BGIOSGA005493 | 55871.32 | 6.38 |
| OsI034802 | BGIOSGA034802 | 81241.76 | 6.63 |
| OsI015975 | BGIOSGA015975 | 79228.81 | 8.79 |

**Supplementary Table 3. Primer and probe sequences used in the study (F, forward primer; R, reverse primer).**

| **qRT-PCR Primers of Genes** | **Sequence (5’-3’)** |
| --- | --- |
| *Os11g08400* (*OsAsp1*) | **F** GCCGACTCACTCTGCACTGAC |
|  | **R** CCGAGGATGCTGTCTACCG |
| *Os03g31630* (*OsSUB29*) | **F** CCTACCGGACTTACATCGTG |
|  | **R** TGACCTGATGCTTCCGATTC |
| *Os07g39020* (*OsSUB53*) | **F** GTTTCCCGATCCCGTTCTAC |
|  | **R** CCATCTTGGTGTACAGGGAC |
| *Os09g30250* (*OsSUB58*) | **F** CGTCATCATTGGCAATCTAGAC |
|  | **R** GTACCCCTTGTTGAAGTACCTC |
| *Os12g23980* (*OsSUB63*) | **F** GCGAGAGGAGAGAAACAACTC |
|  | **R** TTGCCGCAGAAGGTAGTG |
| *OsActin3* | **F** AGTGGTCGTACAACAGGTA |
|  | **R** TCTTCATTAGGCAGTCAGT |

**Supplementary Table 4. Update ID of subtilisin gene family members in *O. sativa* ssp. *japonica.***

| **OLD** (2006) | **NEW** (2022) |
| --- | --- |
| LOC_Os01g17160 (*OsSUB1*) | Delete |
| LOC_Os06g06800 (*OsSUB48*) | LOC_Os06g06820 (*OsSUB48*) |
| LOC_Os01g58260 (*OsSUB6*) | LOC_Os01g58240 (*OsSUB5*) |
| LOC_Os04g03060 (*OsSUB34*) | LOC_Os04g03050 (*OsSUB34*) |
| LOC_Os04g03800 (*OsSUB37*) | LOC_Os04g03796 (*OsSUB37*) |
| LOC_Os04g48420 (*OsSUB45*) | LOC_Os04g48416 (*OsSUB45*) |
| - | LOC_Os09g30458 (*OsSUB64*) (Add) |

**Supplementary Table 5. *OsSUB* expression patterns in different rice tissues from the RNA-seq library.**

| **Locus_id** | **Name** | **Leaves-20 days** | **Post-emergence inflorescence** | **Pre-emergence inflorescence** | **Anther** | **Pistil** | **Seed-5 DAP** | **Embryo- 25 DAP** | **Endosperm- 25 DAP** | **Seed- 10 DAP** | **Shoots** |
| --- | --- | --- | --- | --- | --- | --- | --- | --- | --- | --- | --- |
| LOC_Os02g10520 | OsSub12 | 1.15161 | 11.3842 | 26.535 | 0.777675 | 3.04929 | 0.643298 | 0 | 0 | 0 | 1.47927 |
| LOC_Os01g58270 | OsSub7 | 0 | 0 | 0 | 0.19947 | 0 | 0 | 0 | 0 | 0 | 0.349324 |
| LOC_Os06g41880 | OsSub51 | 0.212946 | 2.462 | 2.49108 | 1.42614 | 2.15757 | 1.21299 | 2.38198 | 0 | 0.403075 | 0 |
| LOC_Os01g52750 | OsSub3 | 5.21135 | 15.3593 | 3.73515 | 0.586392 | 5.97076 | 7.43076 | 0.365472 | 0 | 0 | 3.58582 |
| LOC_Os03g40830 | OsSub30 | 23.7395 | 31.815 | 109.084 | 1.92959 | 23.0172 | 24.1365 | 1.90259 | 1.77128 | 0.388134 | 13.426 |
| LOC_Os02g53860 | OsSub22 | 42.029 | 31.7081 | 58.6112 | 4.45878 | 26.9134 | 3.10838 | 0.375906 | 0 | 0 | 8.71752 |
| LOC_Os08g35090 | OsSub56 | 0 | 6.24337 | 23.6695 | 0.179403 | 13.3964 | 12.1236 | 36.2208 | 0.879981 | 2.79298 | 0.76947 |
| LOC_Os12g23980 | OsSub63 | 1.98373 | 0 | 0 | 0 | 2.04629 | 47.9615 | 0.254325 | 0 | 0 | 0 |
| LOC_Os04g48416 | OsSub45 | 0 | 30.1015 | 165.498 | 0.600114 | 65.3446 | 29.1228 | 24.3417 | 7.29405 | 6.01686 | 7.8361 |
| LOC_Os04g48416 | OsSub45 | 0 | 30.1015 | 165.498 | 0.600114 | 65.3446 | 29.1228 | 24.3417 | 7.29405 | 6.01686 | 7.8361 |
| LOC_Os06g06810 | OsSub49 | 4.26953 | 6.24061 | 7.69787 | 7.33894 | 7.43556 | 4.31735 | 6.38878 | 0.94503 | 2.33075 | 4.71689 |
| LOC_Os04g10360 | OsSub40 | 0 | 17.0747 | 0 | 18.5794 | 7.10734 | 0 | 0 | 0 | 0 | 0 |
| LOC_Os02g17000 | OsSub14 | 0 | 0 | 0 | 0 | 0 | 0 | 0 | 0 | 0 | 4.94531 |
| LOC_Os02g16940 | OsSub13 | 1.50538 | 0 | 0 | 0 | 0 | 0 | 0 | 0 | 0 | 3.42488 |
| LOC_Os01g64860 | OsSub11 | 0 | 0 | 52.0954 | 0 | 0.357969 | 1.35613 | 0 | 0 | 0 | 7.84068 |
| LOC_Os10g25450 | OsSub60 | 0 | 14.4608 | 28.6644 | 0.256137 | 26.3112 | 1.03926 | 1.25185 | 0 | 0 | 0 |
| LOC_Os02g53910 | OsSub23 | 0 | 0 | 0 | 0 | 0 | 0 | 0 | 0 | 0 | 0 |
| LOC_Os02g17080 | OsSub16 | 0 | 0 | 0 | 0 | 0 | 0 | 0 | 0 | 0 | 0 |
| LOC_Os06g48650 | OsSub52 | 0.556695 | 15.9133 | 22.0499 | 3.2778 | 29.9716 | 20.4141 | 54.7018 | 1.90543 | 4.39914 | 1.58248 |
| LOC_Os07g39020 | OsSub53 | 0 | 23.585 | 0 | 0 | 323.987 | 258.154 | 0 | 0 | 0 | 0 |
| LOC_Os10g38080 | OsSub61 | 8.36295 | 60.5179 | 55.8536 | 28.2987 | 74.9935 | 26.0319 | 35.7598 | 2.13571 | 2.85581 | 0.738608 |
| LOC_Os05g30580 | OsSub46 | 0 | 2.00102 | 0 | 0 | 0 | 0 | 0 | 0 | 0 | 0 |
| LOC_Os04g45960 | OsSub42 | 0.272559 | 1.49914 | 84.7195 | 0.685765 | 5.42811 | 1.84547 | 2.50236 | 0.472078 | 1.35109 | 1.03181 |
| LOC_Os03g13930 | OsSub28 | 2.71647 | 16.0067 | 25.0448 | 3.75781 | 51.5263 | 20.7269 | 40.9585 | 2.27326 | 4.21693 | 1.666 |
| LOC_Os04g03100 | OsSub35 | 0 | 0 | 0 | 0 | 0 | 0 | 0 | 0 | 0 | 0 |
| LOC_Os11g15520 | OsSub62 | 0.434327 | 0.61766 | 0.841374 | 0 | 0.328657 | 0 | 1.07824 | 0 | 0 | 0 |
| LOC_Os02g53850 | OsSub21 | 1.15556 | 0 | 0 | 0 | 0 | 0 | 0 | 0 | 0 | 0 |
| LOC_Os03g04950 | OsSub26 | 0 | 1.02659 | 1.82375 | 0 | 0 | 0 | 0 | 0 | 0 | 0 |
| LOC_Os02g44590 | OsSub20 | 0 | 4.13518 | 0.192143 | 42.6038 | 53.4761 | 0 | 0 | 0 | 0 | 0 |
| LOC_Os09g30458 | OsSub64 | 5.19432 | 8.03496 | 8.27095 | 10.9057 | 4.39817 | 1.11114 | 0 | 0 | 0 | 0.4806 |
| LOC_Os03g31630 | OsSub29 | 0 | 0 | 0 | 0 | 1.75016 | 13.4247 | 0 | 0 | 0 | 0 |
| LOC_Os02g17090 | OsSub17 | 0 | 0 | 0 | 0 | 0 | 0 | 0 | 0 | 0 | 1.50851 |
| LOC_Os04g47150 | OsSub43 | 0 | 4.61928 | 0 | 246.204 | 0.57291 | 0 | 0 | 0 | 0 | 0 |
| LOC_Os03g02750 | OsSub25 | 12.2524 | 117.244 | 136.528 | 0.720637 | 264.489 | 46.1286 | 19.2682 | 0.500709 | 0.657832 | 3.07644 |
| LOC_Os09g26920 | OsSub57 | 1.14187 | 3.64081 | 6.26627 | 0 | 1.76995 | 1.32259 | 1.83612 | 0 | 0 | 1.37107 |
| LOC_Os01g58290 | OsSub9 | 1.79634 | 2.69894 | 1.02264 | 5.35588 | 1.98191 | 7.46933 | 3.48139 | 0.465145 | 0.983 | 13.8935 |
| LOC_Os04g02960 | OsSub32 | 0 | 0 | 0 | 0 | 0 | 0 | 0 | 0 | 0 | 0 |
| LOC_Os02g44520 | OsSub19 | 11.6585 | 18.4164 | 34.2341 | 7.78221 | 15.0801 | 16.6888 | 15.4936 | 2.97899 | 8.98117 | 28.5972 |
| LOC_Os04g03850 | OsSub39 | 0 | 0 | 0 | 0 | 0 | 0 | 0 | 0 | 0 | 0 |
| LOC_Os02g53970 | OsSub24 | 0 | 186.902 | 3.73025 | 0 | 0.377878 | 0 | 0 | 0 | 0 | 0 |
| LOC_Os05g36010 | OsSub47 | 4.61616 | 13.4455 | 2.58831 | 0 | 7.3662 | 0.986636 | 0 | 0 | 0 | 5.09677 |
| LOC_Os06g40700 | OsSub50 | 0 | 37.2411 | 0 | 62.5729 | 0 | 0 | 0 | 0 | 0 | 0 |
| LOC_Os04g35140 | OsSub41 | 3.22115 | 3.61235 | 2.4837 | 18.9949 | 2.75893 | 1.71531 | 0.40563 | 1.09383 | 0.648528 | 3.65668 |
| LOC_Os04g03810 | OsSub38 | 0 | 0.945157 | 11.8165 | 0.220807 | 1.1354 | 0.424093 | 14.4044 | 0 | 0 | 3.20016 |
| LOC_Os02g17150 | OsSub18 | 0 | 0 | 0 | 0 | 0 | 0 | 0 | 0 | 0 | 0 |
| LOC_Os04g03050 | OsSub34 | 0.688252 | 36.0855 | 10.4793 | 0 | 0.515712 | 0 | 0 | 0 | 0 | 0 |
| LOC_Os01g56320 | OsSub4 | 0.232526 | 3.67497 | 19.035 | 0.164156 | 20.2907 | 3.36482 | 16.8397 | 0.261869 | 0.554258 | 4.74897 |
| LOC_Os04g03796 | OsSub37 | 5.98394 | 4.57992 | 7.14858 | 2.92077 | 5.36476 | 3.11846 | 6.11957 | 0.446925 | 1.69921 | 42.8275 |
| LOC_Os04g03710 | OsSub36 | 0 | 0 | 0 | 0 | 0 | 0 | 0 | 0 | 0 | 0 |
| LOC_Os04g47160 | OsSub44 | 0 | 0 | 0 | 0 | 0 | 0 | 0 | 0 | 0 | 0 |
| LOC_Os07g48650 | OsSub54 | 0 | 0.276406 | 0 | 0 | 7.35891 | 0 | 0 | 0 | 0 | 0 |
| LOC_Os02g17060 | OsSub15 | 0 | 0 | 0 | 0 | 0 | 0 | 0 | 0 | 0 | 1.33434 |
| LOC_Os08g23740 | OsSub55 | 0 | 137.898 | 0 | 251.799 | 1.16934 | 0 | 0 | 0 | 0 | 0 |
| LOC_Os01g50680 | OsSub2 | 0 | 4.04294 | 2.0219 | 0.127901 | 0 | 0 | 0 | 0 | 0 | 0 |
| LOC_Os03g06290 | OsSub27 | 0.7914 | 1.11175 | 5.90765 | 0.407099 | 1.07422 | 1.62096 | 0.393608 | 0.517598 | 1.7312 | 1.17708 |
| LOC_Os01g58240 | OsSub5 | 4.1528 | 1.68809 | 1.57825 | 2.19294 | 2.21199 | 2.11434 | 1.74275 | 0.210246 | 0.606311 | 4.48669 |
| LOC_Os01g58280 | OsSub8 | 3.34754 | 0.452105 | 1.52306 | 8.85283 | 0.730604 | 0.600665 | 0.255805 | 0 | 0 | 0 |
| LOC_Os09g30250 | OsSub58 | 0 | 0.488956 | 2.94052 | 0.613002 | 2.81397 | 13.9376 | 1.84694 | 0.353078 | 0 | 2.77792 |
| LOC_Os03g55350 | OsSub31 | 2.01666 | 92.1466 | 233.763 | 25.1945 | 204.285 | 198.727 | 248.012 | 16.4503 | 38.89 | 10.4309 |
| LOC_Os09g36110 | OsSub59 | 0.490035 | 1.02258 | 6.47571 | 0.474825 | 1.79673 | 0.278292 | 0.903936 | 0 | 0.184563 | 2.08668 |
| LOC_Os01g64850 | OsSub10 | 0 | 0 | 0.266872 | 0 | 0 | 0 | 0 | 0 | 0 | 0 |
| LOC_Os06g06790 | OsSub48.1 | 1.81793 | 8.38744 | 14.8255 | 1.5764 | 17.4365 | 14.9835 | 11.51 | 1.59394 | 3.25985 | 5.3934 |
